# Skin capillary endothelial cells form a network of spatiotemporally conserved Ca^2+^ activity

**DOI:** 10.1073/pnas.2519708123

**Published:** 2026-06-23

**Authors:** Anush Swaminathan, David G. Gonzalez, Catherine Matte-Martone, Fei Xu, Deandra Simpson, Jessica L. Moore, Zhongqi Lin, Ushnish Rana, David Monedero-Alonso, Julia J. Mack, Chen Yuan Kam, Valentina Greco

**Affiliations:** ^a^Department of Genetics, Yale School of Medicine, New Haven, CT 06510; ^b^ Division of Cardiology, Department of Medicine, University of California, Los Angeles, CA 90095; ^c^ Molecular Biology Institute, University of California, Los Angeles, CA 90095; ^d^ Division of Dermatology, Department of Medicine, University of California, Los Angeles, CA 90095; ^e^ Broad Stem Cell Research Center, University of California, Los Angeles, CA 90095; ^f^ Department of Molecular, Cell and Developmental Biology, University of California, Los Angeles, CA 90095; ^g^ Departments of Cell Biology and Dermatology, Yale Stem Cell Center, Yale Cancer Center, Yale School of Medicine, New Haven, CT 06510; ^h^HHMI, Chevy Chase, MD 20815

**Keywords:** vascular biology, Ca^2+^ signaling, live imaging

## Abstract

Ca^2+^ signaling and its regulation are important for endothelial cell (EC) function and signaling. Yet, the spatiotemporal organization of Ca^2+^ activity and its regulation across a vascular plexus is poorly understood in an in vivo mammalian context. To overcome this gap in knowledge, we developed an intravital imaging approach to resolve Ca^2+^ activity with single-cell resolution in skin vasculature of adult mice via multiphoton microscopy. Here, we tracked thousands of Ca^2+^ events in the skin capillary plexus during homeostasis and observed signaling heterogeneity between ECs, with just over half displaying Ca^2+^ activity at any given time. Longitudinal tracking of the same mice revealed that the same capillary ECs maintain Ca^2+^ activity over days to weeks. Interestingly, activity dynamics, such as frequency and event duration, are not conserved at a single-cell level but are maintained at an EC population level. Molecularly, conditional deletion of the gap junction protein Connexin 43 (Cx43cKO) in ECs leads to a subset of ECs displaying sustained Ca^2+^ activity, biasing signaling dynamics of the whole network toward chronically persistent activity over time. Sustained capillary Ca^2+^ activity results in vascular permeability and flow dysregulation. Last, through pharmacological targeting of known agonists/antagonists, we showed that inhibition of L-type Voltage Gated Ca^2+^ channels non-cell-autonomously restores Ca^2+^ activity, blood flow, and barrier function in Cx43cKO mice. Collectively, our work provides insight into the spatial and temporal characteristics, extent, and regulation of Ca^2+^ activity in skin capillaries of live mice.

## Significance

Ca^2+^ signaling in mammalian endothelial cells (ECs) regulates blood flow, force-sensing, and vessel permeability. While past studies have investigated EC Ca^2+^ signaling in 2D monolayers and the 3D mouse brain, how Ca^2+^ signaling is organized over space and time across general vasculature remains poorly understood. Here, long-term intravital imaging of the same ECs across 3D skin capillaries of live mice reveals that a conserved EC network participates in Ca^2+^ signaling over time. How this network maintains itself requires communication through gap junction protein Connexin 43 (Cx43). Cx43 loss in ECs leads to increased Ca^2+^ activity, blood flow, and vascular permeability. Notably, chemical inhibition of L-type Ca^2+^ channels on cells within the tissue restores physiological Ca^2+^ activity, flow patterns, and barrier function.

Ca^2+^ is an essential second messenger, mediating tightly regulated signaling pathways to integrate spatial and temporal information across multiple scales, from individual cell transients to multicell patterns (1–5). In vitro, the temporal dynamics of Ca^2+^ activity within cells differentially encodes downstream functions, enabling its versatility as a signaling pathway (6–11). In excitable tissues in vivo, such as basal and lateral amygdala (BLA) after application of a conditioning stimuli, there is considerable overlap in which specific neurons are active over days (12). Moreover, the BLA network displays tightly conserved population-level properties, such as maintenance of the total proportion of Ca^2+^ active neurons over time (12). In nonexcitable tissues in vivo, we previously demonstrated that cells in the epidermal stem cell compartment engage in both local and large-scale Ca^2+^ activity without any stimuli (4). These tissue-wide analyses revealed that all epidermal stem cells display Ca^2+^ activity within 1 d during homeostasis. However, in vivo Ca^2+^ activity, characteristics, and regulation, in other tissues, especially in mammalian systems, remains relatively unexplored.

Endothelial cells (ECs) are specialized squamous cells that line the lumen of all blood vessels (13). The endothelium regulates key functions in the vasculature through Ca^2+^ activity, such as angiogenesis, vessel tone, local blood flow control, mechanotransduction, and barrier integrity (14–19). In vivo work in zebrafish vasculature during development has tracked Ca^2+^ across EC populations and identified spatiotemporally organized Ca^2+^ activity specifically in cells driving angiogenesis (tip cells) (20). These cells also display variable Ca^2+^ temporal dynamics which shape downstream vessel fates (20). Recently, more studies have focused on mouse brain capillaries given their extensive neurovascular coupling and outsized energy and metabolic demands, to identify molecular mechanisms driving Ca^2+^ signaling within individual cells. These include IP_3_R-mediated Ca^2+^ release, and how channels including Kir2.1, TRPV4, TRPA1, and Piezo1 shape Ca^2+^ influx (21–25). Yet we lack a spatiotemporal understanding of Ca^2+^ signaling dynamics during homeostasis across more general, peripheral vascular plexi in their native, unperturbed environment.

Organization of Ca^2+^ activity also involves information exchange between cells, as ECs can coordinate Ca^2+^ activity in response to different homeostatic cues (26–30). While individual ECs can regulate cytoplasmic Ca^2+^ concentrations through a variety of mechanisms, multicellular coordination of Ca^2+^ activity between ECs has largely been studied in the context of gap junction-mediated intercellular communication (31). Gap junctions are intercellular channels that allow for cell–cell communication in all tissues, including the vasculature, through electrical coupling and transfer of second messengers such as IP_3_ (32, 33). Gap junctions in the vascular endothelium can facilitate fast, local propagation of vasodilatory signals and enable efficient neurovascular coupling (26, 34–36). Additionally, gap junction activity can modulate EC function during homeostasis, inflammation, and injury, through ATP release and purinergic signaling pathways (37–39). Evidence for EC coordination during homeostasis, development, and injury indicates that molecular signaling may not only be influenced by direct neighbors but also regulated on a tissue-wide scale (40–44). Gap junctions have been implicated in coordination of Ca^2+^ signaling networks in other tissues, such as the loss of Connexin 43 (Cx43) gap junctions leading to tissue-wide uncoupling of coordination across the epidermal stem cell compartment with repeated, more frequent, and longer signaling within restricted neighborhoods (4). In *Drosophila* lymph glands, inhibiting gap junctions alters the Ca^2+^ signaling frequency and promotes precocious differentiation of blood progenitors (45).

Despite these important insights, it remains difficult to study Ca^2+^ dynamics in an in vivo mammalian system given the technical challenges of tracking ECs and their signaling across space and time. Here, we overcome this challenge by focusing on the skin capillary plexus, due to its critical roles in nutrient and oxygen delivery, and tracking Ca^2+^ dynamics in hundreds of ECs over time through our noninvasive, intravital imaging approach (*SI Appendix*, Fig. S1) (4, 42, 46). We observed extensive Ca^2+^ activity across capillary plexus ECs during homeostasis, with patterns of Ca^2+^ activity conserved spatially within the same cells and temporally at the network level. To probe how the spatiotemporal organization of EC Ca^2+^ activity is coordinated, we conditionally deleted Connexin 43 (Cx43cKO) in ECs and found chronically sustained Ca^2+^ activity, altered blood flow dynamics, barrier dysfunction, and dysregulation of EC temporal coordination across the capillary plexus. Finally, we show that inhibition of L-type Voltage Gated Ca^2+^ channels (VGCCs) can restore Ca^2+^ activity in Cx43cKO ECs, along with blood flow and barrier function. Altogether, this study defines the spatiotemporal organization and regulation of Ca^2+^ activity by a conserved EC network in the skin capillary plexus.

## Results

### Spatiotemporal Analyses of the Skin Capillary Plexus Reveal a Network of Endothelial Cells with Conserved Ca^2+^ Activity over Time.

**Fig. 1.**
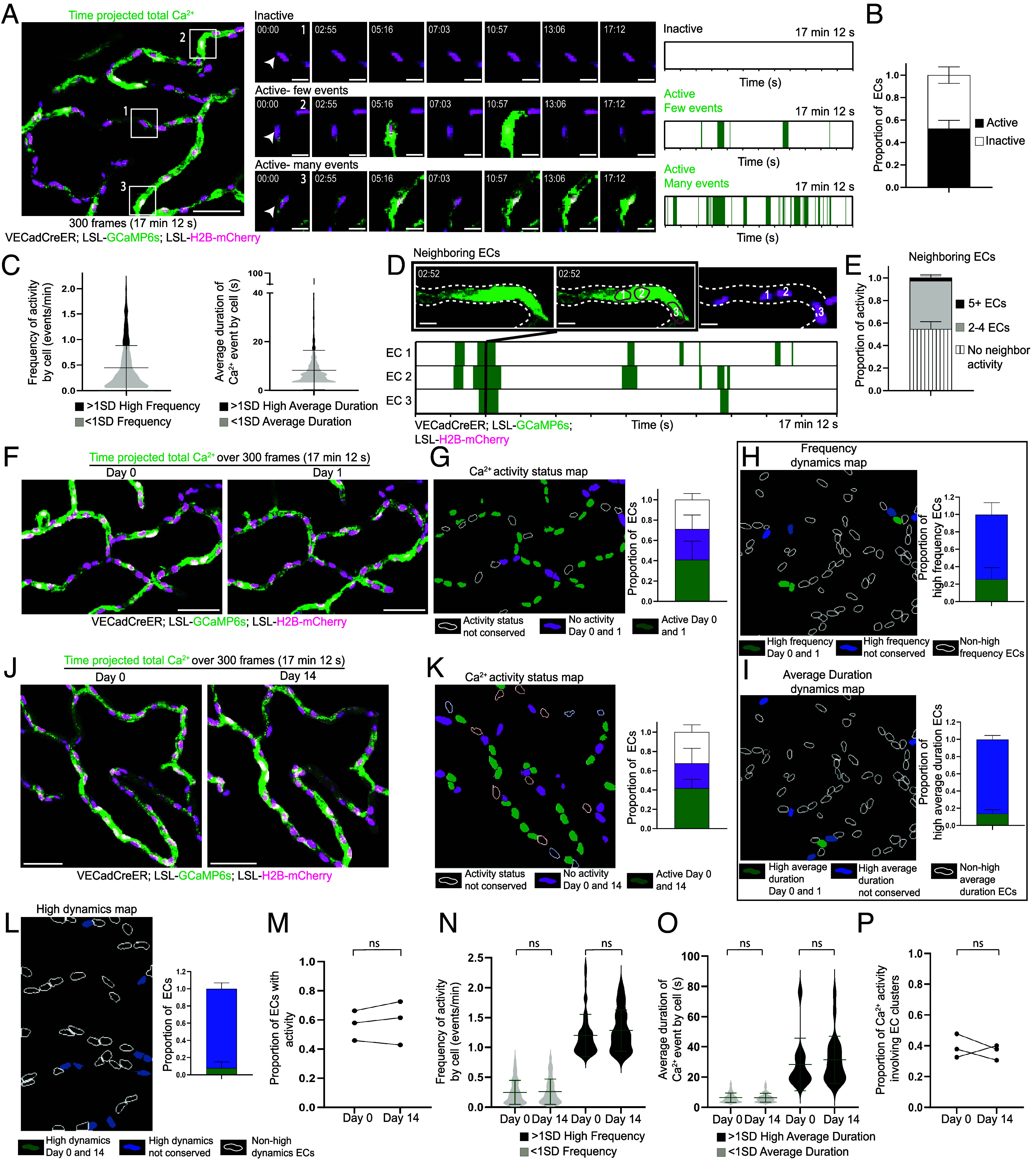
The skin capillary plexus displays Ca^2+^ activity orchestrated by a spatiotemporally conserved network of endothelial cells (*A*, *Left*) Max intensity projection of GCaMP6s signal (green) with H2B-mCherry signal (magenta) from 300 frame (17 min 12 s) recording of skin capillary ECs showing heterogenous Ca^2+^ activity between cells (Scale bar: 50 µm). (*Middle*) *Insets* of three different regions represent differences in Ca^2+^ activity between cells (Scale bar: 10 µm). (*Right*) Ca^2+^ events and their durations over the recording time for each of the ECs displayed in the *Insets* (green represents Ca^2+^ activity) (*B*) Proportion of ECs displaying Ca^2+^ activity (active ECs in black and inactive ECs in white). *n =* 14 regions from four mice. (*C*) Frequency of Ca^2+^ activity per cell (events/min) and average duration (s) of a Ca^2+^ event per cell across 1,765 ECs from four mice. EC groups represented by black (>1 SD high activity) and gray (<1 SD activity) (*D*, *Top*) Ca^2+^ activity occurring simultaneously across three ECs (numbered and drawn in magenta outline matching H2B-mCherry) (Scale bar: 10 µm). (*Bottom*) Ca^2+^ events and their durations over recording time for each EC. Black line across plots indicates the timepoint when all three ECs simultaneously display Ca^2+^ activity. (*E*) Proportion of Ca^2+^ activity involving various EC cluster sizes. *n* = 14 regions from four mice. (*F*) Max intensity projection of GCaMP6s signal with H2B-mCherry signal, with the same regions revisited and recorded 24 h later (Scale bar: 50 µm). (*G*, *Left*) Ca^2+^ activity status map for ECs revisited after 24 h in (*F*). Nonconserved activity status (white outline), inactive both days (magenta) and active both days (green). (*Right*) Proportion of ECs by conservation of their activity status. Chi-square analysis (observed activity 24 h after versus random activity as null hypothesis) *P* < 0.0001. *n =* 7 regions from three mice. (*H* and *I*, *Left*) Dynamics map for ECs revisited after 24 h for conserved high frequency or high average duration behavior on Day 0 and Day 1 (green), not conserved (blue), or not observed (white outline) groups. (*Right*) Conservation of >1 SD dynamics behavior (green) as a proportion of total high frequency or high average duration ECs. *n =* 7 regions from three mice. (*J*) Max intensity projection with the same region revisited and recorded 14 d later (Scale bar: 50 µm). (*K*, *Left*) Ca^2+^ activity status map for ECs revisited after 14 d in (*J*). (*Right*) Conservation of activity status as a proportion of total ECs. Chi-square (observed activity 14 d after versus random activity as null hypothesis) *P* < 0.0001. *n =* 8 regions from three mice. (*L*, *Left*) Dynamics map for ECs revisited after 14 d for conserved >1 SD high dynamics (including both high frequency and high average duration). (*Right*) Conservation of >1 SD high dynamics behavior (green) as a proportion of total high frequency and high average duration ECs. *n =* 8 regions from three mice. (*M*) Proportion of active ECs from three mice on Day 0 and Day 14; ns: *P* > 0.05, paired *t* test, *n =* 457 total cells from three mice. (*N* and *O*) Frequency of Ca^2+^ activity (events/min) and average duration (s) of Ca^2+^ events per cell on Day 0 and Day 14. Black (>1 SD high activity) and gray (<1 SD activity); ns: *P* > 0.05, unpaired *t* test, *n =* 265 active cells from three mice. (*P*) Proportion of Ca^2+^ activity involving EC clusters from three mice on Day 0 and Day 14; ns: *P* > 0.05, paired *t* test.

Understanding plexus-wide Ca^2+^ activity is fundamental to reveal how ECs coordinate their signaling to achieve proper vascular function. The superficial nature of the skin capillary plexus makes it especially tractable to longitudinal tracking of an in vivo EC population through noninvasive multiphoton microscopy. Here, we aimed to gain a plexus-wide view of Ca^2+^ activity in skin capillaries with single-cell resolution. Toward this goal, we recorded Ca^2+^ activity with simultaneous imaging of Ca^2+^ dynamics and EC nuclei via genetically encoded fluorescent reporters. We used an EC-specific inducible Cre driver under the control of the vascular endothelial cadherin (VECad) promoter to simultaneously recombine a nuclear H2B-mCherry reporter and GCaMP6s, a Ca^2+^ reporter encoding a calmodulin-GFP fusion protein (VECadCreER; Rosa26-CAG-LSL-GCaMP6s; LSL-H2B-mCherry mice induced with 2 mg tamoxifen daily for four consecutive days; *SI Appendix*, Fig. S1*A*). We focused on a simplified skin model devoid of hair follicles, palmoplantar skin, and recorded Ca^2+^ activity in the entire capillary plexus (4 to 5 regions per mouse, with 100 to 150 cells per region across 12 microns in depth and 5 optical sections) over a period of 17 min and 12 s (300 frames at 3.44 s/frame) in adult (2 to 4 mo old) anesthetized mice via two-photon microscopy (*SI Appendix*, Fig. S1). During these timelapse recordings, we observed dynamic and heterogeneous Ca^2+^ activity across the plexus with hundreds of events per region and different levels of Ca^2+^ activity and dynamics between cells (Fig. 1*A* and Movie S1). To quantitatively analyze the dynamics of the observed Ca^2+^ activity, we devised a computational analysis pipeline that segments individual ECs by using the position of the nuclear H2B-mCherry signal as a proxy for the cell body (*SI Appendix*, Fig. S1 *A*–*C*). This enabled us to isolate and analyze changes in GCaMP intensity of each individual cell within the imaging region for the entire duration of the timelapse recording (*SI Appendix*, Fig. S1*C*). The pipeline determines the number and duration of Ca^2+^ events per cell without being confounded by variable baseline Ca^2+^ levels, by counting any activity above a set threshold as an event (>50% change in average MFI above the minimum MFI) (Fig. 1 and *SI Appendix*, Fig. S1*C*). We analyzed 1,765 cells across 4 mice and identified that just over half of ECs (52.4 ± 7.34%) exhibited Ca^2+^ activity during the timeframe of recording (defined as having an “active” cell status, with the others assigned a status of “inactive”; Fig. 1*B*).

Considering the GCaMP6s sensor decays with a half-time of approximately 0.6 to 0.9 s (47, 48), we wanted to test to what extent the chosen 3.44 s/frame imaging parameters may affect our ability to accurately capture EC Ca^2+^ dynamics. Comparing 3.44 s/frame imaging to 0.62 s/frame imaging (using reduced optical sections and at the expense of visualizing network connectivity) showed no significant differences in the percentage of signaling ECs captured (52.4 ± 7.34% at 3.44 s/frame vs. 62.8 ± 13.8% at 0.62 s/frame) and demonstrated similar spread in Ca^2+^ event durations (134.2 s maximum duration at 3.44 s/frame imaging vs. 132.7 s maximum duration at 0.62 s/frame imaging) (*SI Appendix*, Fig. S1 *D* and *E*). Given our goal was to interrogate plexus wide Ca^2+^ dynamics, we applied the 3.44 s/frame imaging parameter to capture the entire 3D capillary plexus (*SI Appendix*, Fig. S1*D*) and quantify signaling activity. Using our pipeline, we found that the majority of active ECs display dynamics within a defined range in both frequency (0.445 ± 0.440 events per minute per cell, encompassing 83.9 ± 7.50% of cells) and average duration (8.36 ± 8.15 s per event per cell, encompassing 91.3 ± 3.84% of ECs) for Ca^2+^ events (Fig. 1*C*). A small population of ECs displayed activity above that range, indicating either high frequency (1.31 ± .351 events/min per cell) or high average duration (29.8 ± 16.4 s per cell) dynamics (Fig. 1*C* and *SI Appendix*, Fig. S2*A*). Last, to understand the prevalence of multicellular activity, we adapted our pipeline to identify neighboring cells displaying Ca^2+^ activity either within the same time frame, or one frame apart, and calculated the number of cells involved (Fig. 1*D* and *SI Appendix*, Fig. S1 *A* and *B*). We found that the most represented cluster size for EC Ca^2+^ activity occurred across 2 to 4 cells (42.8 ± 5.64% of total activity) with a small percentage involving five or more cells (2.97 ± 1.32% of total activity) (Fig. 1*E*). Comparing imaging frequency also showed a similar spread in EC cluster sizes (up to 12 cells at 3.44 s/frame imaging vs. 11 cells at 0.62 s/frame imaging) (*SI Appendix*, Fig. S1*E*).

After we established a quantitative understanding of EC Ca^2+^ dynamics, we next asked how activity status is regulated over longer periods of time. Toward this goal, we leveraged our longitudinal imaging approach that allows us to revisit the same cells over days to weeks. We began with short-term revisits and intriguingly, found that not only was the proportion of active and inactive cells largely conserved after 24 h (58.7 ±14.4% active on Day 0 vs. 52.0 ± 18.8% on Day 1), but activity status was also conserved at the cellular level (71.1 ± 6.03% of cells maintained their activity status after 24 h) (Fig. 1 *F* and *G*, *SI Appendix*, Fig. S2*B*, and Movie S2). We also observed that only 28.9 ± 7.89% of high frequency and 17.0 ± 6.30% of high average duration ECs retained their dynamics after 24 h, indicating that despite maintaining activity status (active vs. inactive), individual ECs largely exhibit different Ca^2+^ dynamics (frequency and average duration) over days (Fig. 1 *H* and *I* and *SI Appendix*, Fig. S2*C*).

Next, we asked whether Ca^2+^ activity status is also conserved over a longer time frame. As our past work has demonstrated that ECs remain positionally stable over weeks in adults (42), we revisited the same regions 2 wk later (*SI Appendix*, Fig. S2*D*). Strikingly, we observed that a majority (67.8 ± 7.56%) of the same ECs retained their activity status (Fig. 1 *J* and *K* and Movie S3) even though their dynamic properties were largely not retained (5.58 ± 6.51% retention of high frequency ECs and 0% retention of high average duration ECs) (Fig. 1*L*). Consistent with shorter term revisits, Ca^2+^ dynamics (frequency and average duration), though not conserved at the cellular level, were maintained over weeks at the network level (Fig. 1 *L*–*O*). Lastly, the proportion of Ca^2+^ activity involving EC clusters was also conserved over weeks (Fig. 1*P*).

Collectively, our findings reveal that Ca^2+^ activity in the skin capillary plexus is dynamic during vascular homeostasis and spatially patterned by an active network of ECs with temporally conserved properties over time.

### Loss of Endothelial Connexin 43 Leads to Chronically Sustained Ca^2+^ Activity and Impairs Long-Term Network Regulation.

Our findings that EC Ca^2+^ properties are conserved at the network level over time raise the question of how this is regulated molecularly. Gap junctions are intercellular channels that have established roles in EC communication and functional coupling (34, 49, 50). We hypothesized that maintenance of the active network is spatially coordinated by gap junctions. Connexin 43 is a gap junction protein both widely expressed in the vasculature, and the most expressed Connexin isoform in isolated skin ECs (*SI Appendix*, Fig. S3 *A*, *i*) (51, 52). To investigate the role of Cx43 in the regulation of network Ca^2+^ activity, we combined our Ca^2+^ reporter with Cx43 mutant mice (Cx43^fl/fl^) to generate EC conditional knockouts (VECadCreER; Rosa26-CAG-LSL-GCaMP6s; LSL-H2B-mCherry; Cx43^fl/fl^ denoted as Cx43cKO) and compared them to littermate controls (VECadCreER; Rosa26-CAG-LSL-GCaMP6s; LSL-H2B-mCherry; Cx43^+/+^).

**Fig. 2.**
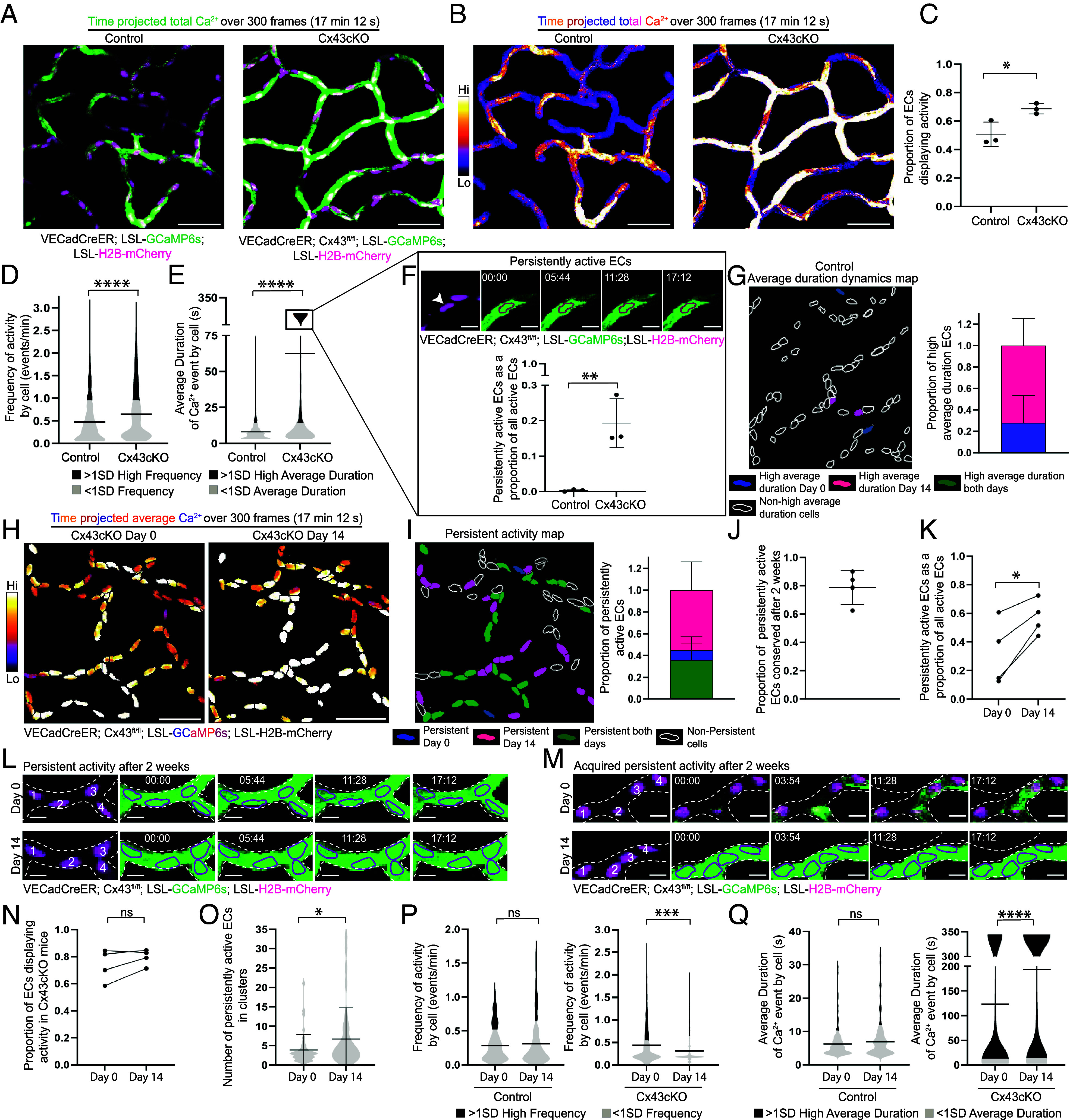
Loss of endothelial Connexin 43 leads to sustained Ca^2+^ dynamics and impairs long-term network regulation. (*A*) Max intensity projection of GCaMP6s signal (green) with H2B-mCherry signal (magenta) from 300 frame (17 min 12 s) recording of skin capillary ECs from control (VECadCreER; Rosa26-CAG-LSL-GCaMP6s; LSL-H2B-mCherry) and Cx43cKO (VECadCreER; Cx43^fl/fl^; Rosa26-CAG-LSL-GCaMP6s; LSL-H2B-mCherry) mice (Scale bar: 50 µm). (*B*) Max intensity projection of GCaMP6s signal from (*A*) represented with fire lookup table. Fire lookup table allows for easier visualization of changes in Ca^2+^ signaling intensity. Color scale indicates GCaMP6s signal over total duration of recording (Scale bar: 50 µm). (*C*) Proportion of active ECs in control and Cx43cKO mice. *P* = 0.0288, unpaired *t* test; *n =* 10 regions from three mice each for control and Cx43cKO. (*D* and *E*) Frequency of Ca^2+^ activity (events/min) and average duration (s) of a Ca^2+^ event per cell respectively for control and Cx43cKO mice; black (>1 SD high activity) and gray (<1 SD activity). *n =* 570 active ECs from three control mice and 837 active ECs from three Cx43cKO mice; *P* < 0.0001 for both, unpaired *t* test. (*F*, *Top*) Representative EC (drawn in magenta outline corresponding with H2B-mCherry label) displaying persistent activity in Cx43cKO mouse (Scale bar: 10 µm). (*Bottom*) Persistently active ECs as a proportion of total active ECs in control and Cx43cKO mice; *P* = 0.009, unpaired *t* test. (*G*, *Left*) Representative average duration cells map of control ECs revisited after 14 d. High average duration ECs on Day 0 (blue), Day 14 (magenta), or both days (green), and non-high-average duration ECs (white outline) (*Right*) Bar graph of high average duration ECs on Day 0, 14, or both days. *n* = 4 regions from three mice (*H*) Average intensity projections of GCaMP6s signal over H2B-mCherry region for single-cell resolution (fire lookup table) in Cx43cKO ECs revisited after 14 d (Scale bar: 50 µm). Color scale indicates average GCaMP6s signal over total duration of recording. Average intensity projection provides visualization of persistently active ECs in white. (*I*, *Left*) Persistent activity map of ECs revisited after 14 d. Persistently active ECs on Day 0 (blue), Day 14 (magenta), or both days (green), and nonpersistently active ECs (white outline). (*Right*) Bar graph of persistently active ECs on Day 0, 14, or both days. *n* = 7 regions from four mice. (*J*) Proportion of persistently active ECs conserved after 14 d among revisited mice. (*K*) Persistently active ECs as a proportion of all active ECs, in mice revisited after 14 d; *P* = 0.023, paired *t* test. *n* = 7 regions from four mice. (*L*) Representative image of ECs (numbered and drawn in magenta outline of H2B-mCherry signal) maintaining persistent activity after 14 d in a Cx43cKO mouse (Scale bar: 10 µm). (*M*) Representative image of ECs gaining persistent activity on Day 14 (Scale bar: 10 µm). (*N*) Proportion of active ECs from Cx43cKO mice on Day 0 and Day 14; ns: *P* > 0.05, *t* test, *n =* 532 total cells from four mice. (*O*) Number of persistently active ECs in clusters, for regions revisited after 14 d; *P* = 0.047, unpaired *t* test. (*P*) Frequency of Ca^2+^ activity (events/min) and average duration (*Q*) of Ca^2+^ events per cell (s) on Day 0 and Day 14 of revisited ECs in control and Cx43cKO mice; black (>1 SD high activity) and gray (<1 SD activity). *P* < 0.0001, unpaired *t* test in Cx43cKO mice; *n* = 399 active cells from four mice. ns = *P* > 0.05, unpaired *t* test in control mice; *n* = 126 active cells from three mice.

Cx43cKO and control mice were induced over four consecutive days (2 mg tamoxifen daily) and then imaged 3 d later (1 wk following the first tamoxifen injection, referred to as Day 0). Surprisingly, we observed an increase in capillary plexus Ca^2+^ activity in Cx43cKO mice compared to controls (Fig. 2 *A* and *B* and Movie S4). This included a significant increase in the proportion of active ECs (68.6 ± 3.71% in Cx43cKO mice vs. 50.8 ± 8.47% in control mice) (Fig. 2*C*). We also observed that the active network in Cx43cKO mice displayed significantly increased frequency (0.646 ± 0.586 events/min per cell in the Cx43cKO mice vs. 0.473 ± 0.467 events/min per cell in control mice) and a significant increase in the average duration of Ca^2+^ events per cell (62.5 ± 119 s per cell in the Cx43cKO mice vs. 8.00 ± 7.33 s per cell in control mice) (Fig. 2 *D* and *E*). Furthermore, we found a group of ECs which displayed significantly long-lasting Ca^2+^ events ranging between 2 and 17 min, a behavior of persistent activity not detected in control mice (19.3 ± 6.51% of active ECs in Cx43cKO mice vs. 0.324 ± 0.305% of active ECs in control mice) (Fig. 2*F*). In contrast to control mice where EC clusters were active simultaneously only for a few frames, Cx43cKO showed “persistently active” EC clusters (61.7 ± 17.3% involved at least two cells), simultaneously active for the entire duration of recording (*SI Appendix*, Fig. S3 *B*–*D*). To understand whether changes in expression of other vascular Connexins (50) may influence Ca^2+^ activity in Cx43cKO mice, we used qPCR to analyze Connexins 37 and 40 expression in isolated skin ECs. We found their transcripts to be similarly expressed in Cx43cKO versus control samples, indicating an absence of compensatory changes in expression (*SI Appendix*, Fig. S3 *A*, *ii*).

We next asked whether spatiotemporal Ca^2+^ activity and network-level dynamics of ECs are conserved over time in Cx43cKO mice, as was observed under physiological conditions. Revisiting the same cells in the same Cx43cKO mice, we found that the activity status of ECs was spatially conserved on a single-cell level after 14 d (78.4 ± 4.84% of ECs retained their status) (*SI Appendix*, Fig. S3 *E* and *F*). Furthermore, persistent Ca^2+^ activity was also conserved on a cellular level after 14 d (78.9 ± 11.5% of persistently active ECs on Day 0 retained their behavior on Day 14) (Fig. 2 *G*–*J* and *L* and Movie S5). While there was no change in the proportion of active ECs after 14 d (Fig. 2*N*), we observed an increase in the proportion of persistently active cells (32.2 ± 22.9% on Day 0 vs. 57.3 ± 12.2% of active ECs on Day 14) (Fig. 2 *K* and *M* and Movie S5). This was accompanied by an increase in the cluster size of ECs with persistent activity (from 3.85 ± 4 cells on Day 0 to 6.73 ± 7.99 cells on Day 14) (Fig. 2*O*). While control mice demonstrated conserved dynamics across the network after 14 d, Cx43cKO mice showed a significant increase in average duration (123 ± 156 s per cell on Day 0 vs. 194 ± 165 s per cell on Day 14) and a significant decrease in frequency (0.445 ± 0.432 events/min per cell on Day 0 vs. 0.319 ± 0.322 events/min per cell on Day 14) per cell, consistent with the observed increase in the number of persistently active cells (Fig. 2 *P* and *Q*).

Our findings show that loss of Cx43 increases plexus-wide Ca^2+^ activity, enriching for a population of persistently active ECs. In addition, we found that loss of Cx43 maintains spatial patterning over weeks, but biases ECs toward longer-lasting Ca^2+^ activity, and accordingly, persistently active ECs become a larger fraction of the vascular plexus over time.

### L-type Voltage-Gated Ca^2+^ Channels (VGCCs) Sustain Elevated EC Ca^2+^ Dynamics Following Loss of Endothelial Connexin 43.

Upon loss of Cx43, we captured an unexpected increase in Ca^2+^ activity across the capillary EC network. To identify what molecular mediators are responsible for this signaling phenotype, we tested a small panel of well-known inhibitors of ion channels involved in Ca^2+^ entry. Specifically, we topically applied GSK219 (TRPV4 inhibitor) (17), Mibefradil (T-type VGCC inhibitor) (53), Nifedipine (L-type VGCC inhibitor) (54), and DMSO vehicle in Cx43cKO mice, and imaged the same capillary regions before and after treatment (within 30 to 60 min) (17, 53, 55). Interestingly, we observed that nifedipine appeared to largely decrease total and persistent Ca^2+^ activity in Cx43cKO mice relative to the other compounds (*SI Appendix*, Fig. S4). In addition to L-type VGCCs, T-type VGCCs (54) are possible other targets of nifedipine. Given their inhibition by Mibefradil did not have any effect on Ca^2+^ activity in Cx43cKO mice, L-type VGCCs specifically emerge as molecular mediators of increased Ca^2+^ activity in ECs after loss of Cx43. To further test for specificity of L-type VGCC inhibition in driving the EC Ca^2+^ reduction after loss of Cx43, we also used a different L-type VGCC inhibitor, Verapamil, and lower doses of nifedipine (10 and 100 times reduced in concentration), all of which decreased Ca^2+^ activity in Cx43cKO mice (*SI Appendix*, Fig. S5*A*).

**Fig. 3.**
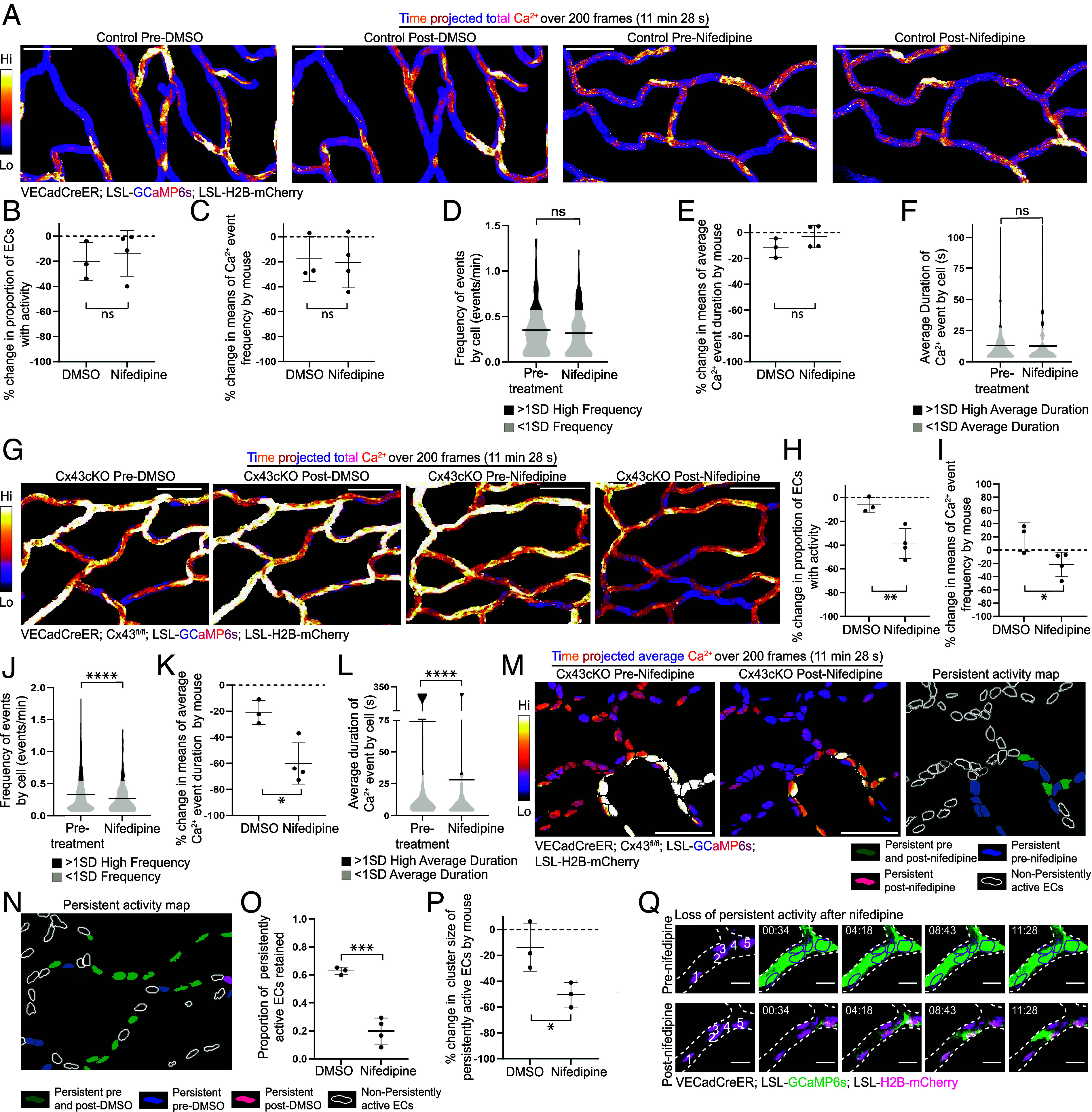
L-type VGCCs regulate sustained Ca^2+^ dynamics after Cx43cKO, but do not affect Ca^2+^ activity under physiological conditions (*A*) Max intensity projection of GCaMP6s signal in control mice before and after treatment with either DMSO or nifedipine. GCaMP6s signal is represented with fire lookup table from 200 frame (11 min 28 s) recording of skin capillary ECs before and after treatment. Fire lookup table allows for easier visualization of changes in Ca^2+^ signaling intensity. Color scale indicates GCaMP6s signal over recording time (Scale bar: 50 µm). (*B*) Percent change in proportion of active ECs in control mice revisited after treatment with either DMSO or nifedipine. ns = *P* > 0.05, unpaired *t* test; *n* = 5 regions from three mice for DMSO treatment and 6 regions from four mice for nifedipine treatment. (*C*) Percent change in frequency (events/min) of Ca^2+^ activity (shown as percentage change of means, refer to *Materials and Methods* under *Statistics and Reproducibility*) in control mice revisited after treatment with either DMSO or nifedipine. ns = *P* > 0.05, unpaired *t* test. (*D*) Frequency of Ca^2+^ activity (events/min) per cell of revisited control mice before and after nifedipine treatment. Black (>1 SD high activity) and gray (<1 SD activity); ns = *P* > 0.05, unpaired *t* test; n = 187 active ECs pretreatment and n = 173 active ECs after treatment, from four mice. (*E*) Percent change in average duration (s) of Ca^2+^ activity in control mice revisited after treatment with either DMSO or nifedipine. ns = *P* > 0.05, unpaired *t* test. (*F*) Average duration (s) of Ca^2+^ events per cell of revisited control mice before and after nifedipine treatment. ns = *P* > 0.05, unpaired *t* test; n = 187 active ECs pretreatment and n = 173 active ECs after treatment, from four mice. (*G*) Max intensity projection of GCaMP6s signal in Cx43cKO mice before and after treatment with either DMSO or nifedipine. (Scale bar: 50 µm). (*H*) Percent change in proportion of active ECs in Cx43cKO mice revisited after treatment with either DMSO or nifedipine. *P* = 0.0097, unpaired *t* test; *n* = 5 regions from three mice for DMSO treatment and 6 regions from four mice for nifedipine treatment. (*I*) Percent change in frequency (events/min) of Ca^2+^ activity in Cx43cKO mice revisited after treatment with either DMSO or nifedipine. *P* = 0.04, unpaired *t* test, for frequency plot. (*J*) Frequency of Ca^2+^ activity (events/min) per cell of revisited Cx43cKO mice before and after nifedipine treatment. *P* < 0.0001, unpaired *t* test; n = 358 active ECs pretreatment and n = 218 active ECs after treatment, from four mice. (*K*) Percent change in average duration (s) of Ca^2+^ activity in Cx43cKO mice revisited after treatment with either DMSO or nifedipine. *P* = 0.013, unpaired *t* test, for frequency plot. (*L*) Average duration of Ca^2+^ activity (s) per cell of revisited Cx43cKO mice before and after nifedipine treatment. *P* < 0.0001, unpaired *t* test; n = 358 active ECs pretreatment and n = 218 active ECs after treatment, from four mice. (*M*, *Left*) Average intensity projections of GCaMP6s signal (fire lookup table) over H2B-mCherry region for single-cell resolution, from regions revisited after nifedipine treatment (Scale bar: 50 µm). Color scale indicates average GCaMP6s signal over total recording time. Average intensity projection improves visualization of persistently active ECs in white. (*Right*) Map of persistently active ECs after nifedipine treatment. Persistently active ECs pre- and post-nifedipine (green), pre-nifedipine (blue), post-nifedipine (magenta), or nonpersistently active ECs (white outline). (*N* and *O*, *Left*) Representative map of persistently active ECs in Cx43cKO mice revisited after treatment with DMSO. (*Right*) Proportion of persistently active ECs retained after DMSO versus nifedipine treatments. *n* = 5 regions from three mice for DMSO and 6 regions from four mice for nifedipine treatment. (*P*) Percent change in cluster size of persistently active ECs revisited after treatment with either DMSO or nifedipine; *P* = 0.038, unpaired *t* test. (*Q*) Representative images of ECs without persistent activity after nifedipine treatment in a Cx43cKO mouse (Scale bar: 10 µm).

Nifedipine is an inhibitor of L-type VGCCs (54–57). To first test whether nifedipine would alter capillary Ca^2+^ activity during homeostasis, we used control (VECadCreER; Rosa26-CAG-LSL-GCaMP6s; LSL-H2B-mCherry) mice and compared the proportion of active ECs, as well as mean values of frequency and average duration by cell per mouse, before and after treatment with nifedipine or DMSO. We did not observe a significant difference in proportion of active ECs (13.7% ± 18.2% decrease after nifedipine treatment vs. 20.2% ± 15.0% decrease after DMSO treatment), frequency (20.6% ± 20.5% decrease after nifedipine treatment vs. 17.8% ± 17.9% decrease after DMSO treatment), or average duration (3.08% ± 8.73% decrease after nifedipine treatment vs. 11.8% ± 7.47 % decrease after DMSO treatment) (Fig. 3 *A*–*C* and *E* and Movie S6). In addition, distributions of Ca^2+^ event frequency and average duration per cell were also not different before or after nifedipine treatment, suggesting that L-type VGCC inhibition does not affect network-wide Ca^2+^ activity in control mice (Fig. 3 *D* and *F*).

Next, to probe deeper into the effect of nifedipine treatment on Ca^2+^ activity in mice lacking EC Cx43, we repeated the experiments utilizing Cx43cKO mice with the H2B-mCherry nuclear reporter. Compared to DMSO treatment, nifedipine treatment led to decreased proportion of active ECs (39.0% ± 12.6% decrease after nifedipine treatment vs. 6.29% ± 6.23% decrease after DMSO treatment), frequency (21.5% ± 18.5% decrease after nifedipine treatment vs. a 20.0% ± 21.5% increase after DMSO treatment), and average duration (72.8% ± 37.0% decrease after nifedipine treatment vs. 29.3% ± 11% decrease after DMSO treatment) (Fig. 3 *G*–*I* and *K* and Movie S7). In addition, the distributions of Ca^2+^ event frequency and average duration per cell were decreased after nifedipine treatment compared to pretreatment (Fig. 3 *J* and *L*). We next analyzed the effects of nifedipine on persistently active cells in Cx43cKO mice and confirmed a decrease in activity after treatment (19.9% ± 9.3% of persistently active cells retained after nifedipine treatment vs. 62.9% ± 2.65% after DMSO treatment) alongside a decrease in mean cluster size of persistently active ECs (50.5% ± 9.53% decrease after nifedipine treatment vs. 18.4% ± 14.0% decrease after DMSO treatment) (Fig. 3 *M*–*Q*).

L-type VGCCs are known to be expressed in mural cells, including pericytes (58–61), but not endothelial cells (51, 52). To confirm this, we isolated skin ECs from Cx43cKO and control mice, extracted mRNA, and showed that neither the L-type VGCC main domain *CACNA1C* or its channel subunit *CACNA2D1* are expressed in ECs, while their expression was verified on isolated pericyte mRNA (*SI Appendix*, Fig. S5 *B* and *C*). All together, these data support a non-cell-autonomous model of regulation whereby L-type VGCCs on other cells in the tissue mediate changes to a more persistent Ca^2+^ activity in capillary ECs after the loss of Cx43, but do not affect EC Ca^2+^ dynamics in control mice.

### Sustained Ca^2+^ Activity after Loss of Endothelial Connexin 43 Leads to Vascular Flow and Barrier Dysfunction.

**Fig. 4.**
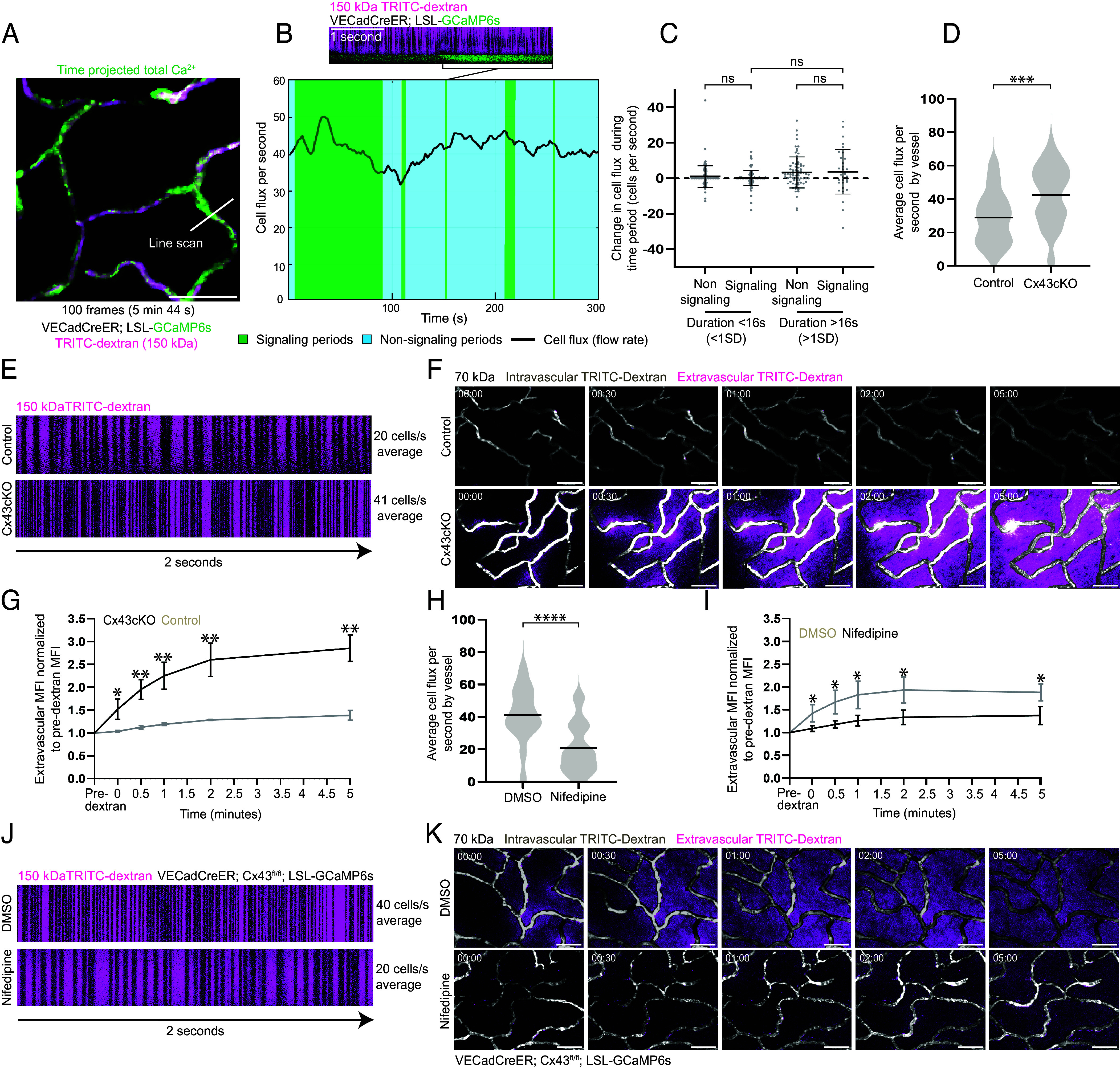
Sustained Ca^2+^ dynamics after Cx43cKO lead to vascular flow and barrier dysfunction. (*A*) Max intensity projection of GCaMP6s signal (green) with 150 kDa TRITC dextran (magenta) from 100 frame (5 min 44 s) recording, with the white line representing a line scanning region (Scale bar: 50 µm). (*B*, *Top*) Representative line scan of a vessel over a 4 s period with Ca^2+^ events (green) and 150 kDa TRITC dextran for flow. Black lines in dextran represent individual cells passing through the line scan (Scale bar: 1 s). (*Bottom*) Graph representing cell flux per second or flow rate during line scanning of an individual vessel over a 5 min period. Flow rate (black line) is represented over signaling (green) and nonsignaling (light blue) time periods. (*C*) Change in flow rate during nonsignaling and signaling time periods (< and >1 SD in duration for each category). ns: *P* > 0.05, unpaired *t* test, *n =* 45 vessels and 148 signaling events from three mice. (*D*) Average cell flux per second by vessel in control and Cx43cKO mice. *P* < 0.001, unpaired *t* test, *n =* 45 vessels from three mice for each group. (*E*) Representative line scans of control and Cx43cKO vessel over a 2 s period. (*F*) Representative single time point images of 70 kDa intravascular dextran (gray) and extravascular dextran (magenta) (Scale bar: 50 µm). (*G*) Line plots of extravascular MFI in control and Cx43cKO mice, normalized to pre-dextran MFI. *P* = 0.020 at 0 min, *P* = 0.003 at 0.5, 1, and 2 mins, and *P* = 0.001 at 5 min, unpaired *t* tests; *n* = three mice each for control and Cx43cKO. (*H*) Average cell flux per second by vessel in Cx43cKO mice treated with DMSO or nifedipine. ns: *P* < 0.0001, unpaired *t* test, *n =* 45 vessels from three mice for each group. (*I*) Line plots of extravascular MFI in DMSO and nifedipine treated Cx43cKO mice, normalized to pre-dextran MFI. *P* = 0.02 at 0 min, *P* = 0.014 at 0.5 min, *P* = 0.017 at 1 min, *P* = 0.015 at 2 min, and *P* = 0.018 at 5 min, unpaired *t* tests; *n =* four mice with nifedipine treatment and three mice with DMSO treatment. (*J*) Representative line scans of Cx43cKO mouse after treatment with DMSO or nifedipine. (*K*) Representative single time point images of 70 kDa intravascular dextran (gray) and extravascular dextran (magenta) after treatment with DMSO or nifedipine (Scale bar: 50 µm).

The elevated Ca^2+^ activity of Cx43cKO ECs over days to weeks was surprising since sustained cytosolic Ca^2+^ has been associated with cell death (62–64). This prompted us to assess EC number and endothelial architecture, but we found no change in cell density or vessel architecture even after revisiting the same cells in Cx43cKO mice after 14 d of sustained Ca^2+^ dynamics (*SI Appendix*, Fig. S6 *A*–*C*). Cx43 has also been shown to affect vascular flow and barrier function (65–72). We first investigated flow rate by injecting 150 kDa tetramethylrhodamine (TRITC) dextran into the vasculature of Cx43cKO and control mice and used line scanning as a method to track the number of cells passing through a region of the vessel over time (cell flux per second) while simultaneously recording Ca^2+^ activity (Fig. 4*A*). We tracked each vessel for a period of 5 min, at 2 s intervals, collecting both flow rate as well as Ca^2+^ signaling events and their durations (Fig. 4*B*). We observed a significant increase in average flow rate per vessel in Cx43cKO mice when compared to controls (42.5 ± 18 cells per second in Cx43cKO mice versus 28.9 ± 15.7 cells per second in control mice) (Fig. 4 *D* and *E* and *SI Appendix*, Fig. S6*E*). Intriguingly, we observed no apparent correlation between individual Ca^2+^ events and changes in local flow in the control mice, and no significant differences between changes in flow rate during signaling and nonsignaling periods while tracking skin capillaries in homeostasis (Fig. 4 *B* and *C* and *SI Appendix*, Fig. S7).

To test the potential impact of Cx43cKO on vessel permeability, we utilized a small size tetramethylrhodamine (TRITC)-dextran (70 kDa), known to be impermeable in skin capillaries during homeostasis (73, 74). We found that within seconds of injection, 70 kDa dextran was found in the interstitial space of skin capillaries of Cx43cKO mice but not in control mice (Fig. 4 *F* and *G*). This is in contrast to the larger size dextran (150 kDa) used to assess flow, which did not leak into the interstitial space in Cx43cKO mice (*SI Appendix*, Fig. S6*D*), indicating Cx43cKO’s barrier phenotype is size-specific and does not reflect an entirely dysfunctional endothelial barrier.

These defects in flow and barrier function prompted the question as to whether they are downstream of elevated Ca^2+^ signaling driven by loss of Cx43. Therefore, we employed nifedipine treatment, since we showed it rescued the Ca^2+^ elevation in Cx43cKO mice (Fig. 3). Excitingly, upon nifedipine treatment, the dysregulated vascular flow was reverted back to physiological patterns (41.2 ± 17.1 cells per second in Cx43cKO mice treated with DMSO versus 20.8 ± 15.3 cells per second in Cx43cKO mice treated with nifedipine) (Fig. 4 *H* and *J* and *SI Appendix*, Fig. S6*F*). Leakage of 70 kDa dextran in the interstitial space of the skin was also significantly reduced with nifedipine treatment, even though we did observe nonspecific reduction of permeability with DMSO treatment (Fig. 4 *I* and *K*).

Altogether, our results demonstrate that sustained Ca^2+^ signaling after loss of EC Cx43 drives vascular flow dysregulation and barrier dysfunction (*SI Appendix*, Fig. S8).

## Discussion

How Ca^2+^ activity is organized, maintained, and regulated across a vascular plexus in its native, unperturbed tissue environment is understudied. Our study longitudinally tracks Ca^2+^ activity of the same in vivo populations of mammalian ECs over minutes to days to weeks, demonstrating unexpected spatiotemporal patterns of endothelial Ca^2+^ activity and dynamics. Our tissue-wide analysis reveals that a network of active cells, conserved over time, orchestrates network Ca^2+^ activity (*SI Appendix*, Fig. S8). Coordinated Ca^2+^ activity as a baseline in adult capillary homeostasis highlights that organized cell networks control Ca^2+^ activity at the tissue-level, and reinforces the notion of the endothelium being a functional syncytium (29, 34, 75). Furthermore, we identified that Cx43 functions to constrain endothelial Ca^2+^ activity within a dynamic range and maintain network signaling dynamics over the long-term (*SI Appendix*, Fig. S8). Our findings elucidate a role for Cx43 as a mediator of cell–cell communication on larger spatial and temporal scales than previously reported. Furthermore, we found that non-cell-autonomous inhibition of L-type VGCCs was able to rescue EC Ca^2+^ signaling patterns, barrier function, and blood flow, revealing that a dynamic range of Ca^2+^ signaling across the EC network allows maintenance of physiological functions (*SI Appendix*, Fig. S8).

In this study, we found and quantified the organization and regulation of homeostatic Ca^2+^ activity in skin vasculature through our unique ability to resolve tissue-wide Ca^2+^ dynamics with single-cell resolution *in an intact, uninjured mouse*. The plexus-wide Ca^2+^ activity in skin ECs in physiological conditions (Fig. 1) is reminiscent of Ca^2+^ activity observed in vivo in other tissues, including mouse skin epidermal stem cells and brain capillaries (4, 17). However, our uncovering of the spatiotemporal conservation of Ca^2+^ activity and its large-scale coordination emerges as critical features of skin capillaries (Fig. 1). Specifically, the finding that EC network activity status is maintained on a cellular level and that the distribution of Ca^2+^ dynamics is conserved on a population level are unique findings across organs and organisms to date. As such, a conceptual advance of our study is our demonstration that Ca^2+^ signaling in capillary endothelial cells is maintained at the network level over time. While large-scale coordination between ECs has been shown in periods of extensive vascular remodeling, such as development and injury (40, 42, 43, 76, 77), our work establishes an unexpected paradigm that Ca^2+^ activity in capillaries is both orchestrated at a tissue-wide scale and is nonrandom in its spatial patterning. Heterogeneity of EC Ca^2+^ activity, which is conserved at a single-cell level over days to weeks, also suggests cellular heterogeneity in the same vascular compartment. This builds upon findings of EC heterogeneity in the same vascular compartment, across the vascular tree, and between organs, as a way for the endothelium to adapt to local environments (73, 78–85). Our findings may further indicate differences in the microenvironment and/or decision-making on a cell-to-cell basis in a capillary plexus.

We find that the gap junction protein Cx43 regulates the active network on a temporal scale by influencing network coordination and maintenance of Ca^2+^ dynamics over time (Fig. 2). Gap junctions and Connexins have been described as intercellular channels that allow for local cell–cell communication in all tissues (32, 33). However, after loss of Cx43 in ECs, there is a loss of temporal maintenance of population-level behaviors that leads to increasing proportion of persistent signaling ECs over weeks, emphasizing a novel long-range, tissue-level regulatory role of Cx43 in temporal maintenance of plexus-wide EC Ca^2+^ signaling.

Ca^2+^ activity in the Cx43cKO capillary plexus converges toward more persistent Ca^2+^ activity without any cell death, while maintaining similar proportions of active ECs (*SI Appendix*, Fig. S6). Given the role of elevated, sustained cytosolic Ca^2+^ in apoptosis and cell death (62–64), we anticipate that other mechanisms, including the protective effects of PMCA (Plasma Membrane Ca^2+^-ATPase) and SERCA (Sarco/Endoplasmic Reticulum Ca^2+^ ATPase) in reducing Ca^2+^ overload to promote cell survival, might function to keep high concentrations of intracellular Ca^2+^ in check (86, 87). The phenotype of persistently active skin capillary ECs in Cx43cKO mice (Fig. 2) also contrasts with published in vitro work in ECs, where pharmacological pan-inhibition of gap junctions, or genetic knockout of different Connexin isoforms, decreases Ca^2+^ activity (26, 29, 35, 88–91). Our data may indicate a more complex regulation of EC Ca^2+^ signaling with the vasculature structurally intact. Moreover, ex vivo approaches have shown that Ca^2+^ activity and spread recorded between adjacent alveolar capillaries is abolished in both EC-specific Cx43 knockout mice and mimetic peptide blocking of Cx43 function (67). We characterized Ca^2+^ signaling across the global vasculature in a native, unperturbed context, which may explain the differences in our findings compared to ex vivo models that more closely mimic injury contexts. Prior to this study, Ca^2+^ activity has not been clearly visualized in vivo after the loss of gap junctions. However, the reported vascular phenotypes in studies involving endothelial conditional deletion of Cx43, Cx37, and Cx40 include sustained vessel dilation and increase of plasma nitric oxide levels (34, 71). These phenotypes are associated with an increase in EC Ca^2+^ activity, and therefore we believe that the differences we observed in skin ECs compared to previously described systems upon loss of Cx43 reflect a combination of tissue-specificity, the physiological, native context, and the unperturbed nature of the vascular architecture.

The interplay we observed between L-type VGCC activity and Cx43 dysregulation (Fig. 3) provides insight into how heterotypic cell interactions can reshape the Ca^2+^ landscape. We propose a model whereby the loss of Cx43 in ECs leads to enhanced L-type VGCC activity on vessel-associated cells, which then non-cell-autonomously regulate EC Ca^2+^ dynamics. Cx43 gap junctions have been implicated in regulating membrane potential through electrical coupling and ion exchange functions (92, 93). In this way, loss of Cx43 in ECs could influence membrane potential and subsequent L-type VGCC activation in neighboring cells, which then through “inside out” signaling mechanisms or paracrine factors may influence EC Ca^2+^ signaling (31, 94–96). Pericytes are a possible candidate as they are known to be coupled electrically with ECs through gap junctions (97–99), can influence EC Ca^2+^ activity in other tissues (58–61, 97–100), and are widespread in skin (*SI Appendix*, Fig. S5*C*); although we cannot rule out tissue-resident macrophages (101) and capillary-associated fibroblasts (102). Another possibility is that the drug acts on L-type VGCCs in smooth muscle cells (SMCs) on the upstream arterioles (103) that shape blood flow into the capillaries. Future work is needed to fully understand the role of voltage gated channels in regulating function of the skin capillary plexus.

Our finding of sustained Ca^2+^ signaling after loss of Cx43 leading to flow and barrier dysfunction indicates that a set dynamic range of Ca^2+^ signaling across the network is necessary for homeostatic maintenance of vascular function, which when exceeded, leads to global flow dysregulation and barrier dysfunction. Ca^2+^ signaling in brain capillaries has been described to drive local blood flow changes (17, 23, 24). Our findings that homeostatic Ca^2+^ signaling is not correlated with apparent blood flow changes may indicate tissue-specific differences in Ca^2+^ regulation of flow and emphasize differences between central vasculature and regulation of general, peripheral vascular beds. In regard to vascular permeability, the direct role of elevated cytosolic Ca^2+^ in disrupting endothelial barrier integrity through RhoA-ROCK1 activation, VE-Cadherin junction disassembly, and cytoskeletal changes has been explored in past studies (104–108). We wonder whether similar mechanisms may drive changes in vascular permeability after sustained Ca^2+^ elevation in the skin vasculature. Increased shear stress can also lead to EC junctional changes (109), and so the reduction in permeability after L-type VGCC inhibition in Cx43cKO mice may be directly influenced by the decrease in vascular flow rate.

In conclusion, our work provides unprecedented spatial and temporal intravital imaging at single-cell resolution to define the spatiotemporally conserved networks of capillary EC Ca^2+^ signaling and reveals that vascular function is compromised when this signaling is altered.

## Materials and Methods

Detailed experimental procedures are described in the *SI Appendix*. Adult mice with an EC-specific Ca^2+^ sensor were anesthetized, their palmoplantar skin was imaged using 2-photon microscopy, and data were analyzed through new quantitative pipelines. For each topical treatment, capillary regions were imaged prior to treatment, the skin was treated with the drug dissolved in DMSO, and then the same regions were revisited 30 to 60 min after treatment. Vascular flow and permeability were assessed through retroorbital injections of different sized dextrans. All statistical analyses were conducted using Graphpad Prism.

## Supplementary Material

Appendix 01 (PDF)

Movie S1. **EC Ca^2+^ activity in skin capillaries is widespread and heterogenous during homeostasis**. Timelapse of GCaMP6s signal (green) and H2B-mCherry (magenta) from 300 frame (17 minutes 12 s) recording of skin capillary ECs, imaged at a frame rate of 3.44 s/frame (scale bar: 20 μm). Timelapse is followed by a max intensity projection of GCaMP6s signal represented with fire lookup table. Color scale indicates GCaMP6s signal over recording time.

Movie S2. **EC Ca^2+^ activity status is conserved on a single-cell level after 24 hours**. *Left*: Timelapse of GCaMP6s signal (green) and H2B-mCherry (magenta) from 300 frame (17 minutes 12 s) recording of skin capillary ECs on baseline Day 0, imaged at a frame rate of 3.44 s/frame (scale bar: 20 μm). Right: Timelapse of region from Day 0 revisited after 24 hours. Timelapse is followed by a max intensity projection of GCaMP6s signal for Day 0 and Day 1 represented with fire lookup table. Color scale indicates GCaMP6s signal over recording time.

Movie S3. **EC Ca^2+^ activity status is conserved on a single-cell level after 14 days**. *Left*: Timelapse of GCaMP6s signal (green) and H2B-mCherry (magenta) from 300 frame (17 minutes 12 s) recording of skin capillary ECs on baseline Day 0, imaged at a frame rate of 3.44 s/frame (scale bar: 20 μm). Right: Timelapse of region from Day 0 revisited after 2 weeks. Timelapse is followed by a max intensity projection of GCaMP6s signal for Day 0 and Day 14 represented with fire lookup table. Color scale indicates GCaMP6s signal over recording time.

Movie S4. **Cx43cKO leads to sustained EC Ca^2+^ activity**. *Left*: Timelapse of GCaMP6s signal (green) and H2B-mCherry (magenta) from 300 frame (17 minutes 12 s) recording of skin capillary ECs from control mice, imaged at a frame rate of 3.44 s/frame (scale bar: 20 μm). *Right*: Timelapse of capillary ECs from Cx43cKO mice. Timelapse is followed by a max intensity projection of GCaMP6s signal for control and Cx43cKO mice, represented with fire lookup table. Color scale indicates GCaMP6s signal over recording time.

Movie S5. **Cx43cKO leads to increase in persistently active ECs after 2 weeks**. *Left*: Timelapse of GCaMP6s signal (green) and H2B-mCherry (magenta) from 300 frame (17 minutes 12 s) recording of skin capillary ECs from Cx43cKO mice on baseline Day 0, imaged at a frame rate of 3.44 s/frame (scale bar: 20 μm). *Right*: Timelapse of region from Day 0 revisited after 2 weeks, on Day 14. Timelapse is followed by an average intensity projection of GCaMP6s signal for Cx43cKO mice on Day 0 and Day 14, represented with fire lookup table. Average intensity projections allow better visualization of persistently active regions in white. Color scale indicates GCaMP6s signal over recording time.

Movie S6. **L-type VGCC inhibition does not affect EC Ca^2+^ activity in control mice**. *Top Left*: Timelapse of GCaMP6s signal (green) and H2B-mCherry (magenta) from 200 frame (11 minutes 28 s) recording of skin capillary ECs from control mice prior to DMSO treatment, imaged at a frame rate of 3.44 s/frame (scale bar: 20 μm). *Top Right*: Timelapse of revisited region after DMSO treatment. Bottom Left: Timelapse of ECs prior to nifedipine treatment. Bottom *Right*: Timelapse of revisited region after nifedipine treatment. All timelapses are followed by max intensity projection of GCaMP6s signal represented with fire lookup table. Color scale indicates GCaMP6s signal over recording time.

Movie S7. **L-type VGCC inhibition decreases EC Ca^2+^ activity after Cx43cKO**. *Top Left*: Timelapse of GCaMP6s signal (green) and H2B-mCherry (magenta) from 200 frame (11 minutes 28 s) recording of skin capillary ECs from Cx43cKO mice prior to DMSO treatment, imaged at a frame rate of 3.44 s/frame (scale bar: 20 μm). *Top Right*: Timelapse of revisited region after DMSO treatment. *Bottom Left*: Timelapse of ECs prior to nifedipine treatment. *Bottom Right*: Timelapse of revisited region after nifedipine treatment. All timelapses are followed by max intensity projection of GCaMP6s signal represented with fire lookup table. Color scale indicates GCaMP6s signal over recording time.

## Data Availability

Coding scripts, all raw data (timelapses, images), source datasheets for figure quantifications data have been deposited in Dataverse (https://dataverse.yale.edu/dataverse/skinendothelialcalcium) (110). Other data are included in the article and/or supporting information.
