# Supplementary material for: Skin capillary endothelial cells form a network of spatiotemporally conserved Ca2+ activity
Source: Proc Natl Acad Sci U S A. 2026 Jun 23;123(26):e2519708123. doi: 10.1073/pnas.2519708123 (PMC13320711; doi:10.1073/pnas.2519708123)
Supplement: Supplementary file 1 — Appendix 01 (PDF) [file pnas.2519708123.sapp.pdf]

Supporting Information for

Skin capillary endothelial cells form a network of spatiotemporally conserved Ca^{2+} activity.

Anush Swaminathan, David G. Gonzalez, Catherine Matte-Martone, Fei Xu, Deandra Simpson, Jessica L. Moore, Zhongqi Lin, Ushnish Rana, David Monedero-Alonso, Julia J. Mack*, Chen Yuan Kam*, Valentina Greco*

*Co-correspondence to: Valentina Greco, valentina.greco@yale.edu, Chen Yuan Kam chenyuankam@mednet.ucla.edu, Julia J. Mack, jmack@mednet.ucla.edu

This PDF file includes:

Supporting text
Figures S1 to S8
Legends for Movies S1 to S7
SI References

Supporting Information Text

Limitations of the study

In this study, we investigated the spatiotemporal characteristics and regulation of tissue-level Ca^{2+} activity across capillary ECs *in vivo*, focusing on the skin. One consideration is using a pan-endothelial driver (VECadherin promoter) that does not allow us to discern EC heterogeneity or perturb specific subpopulations of ECs. Drug treatment absorption into the skin can also be variable. This issue was addressed by assessing multiple regions of the plexus within the same mice before and after treatments.

Supplementary Methods and Materials

Mice

VECadCreER (1) mice were obtained from Ralf Adams (Max Planck, Muster, Germany), *Cx43*$^{fl/fl}$ (2), *Rosa26-CAG-LSL-GCaMP6s (3)*, *Rosa26-CAG-LSL-H2B-mCherry (4)*, *aSMA-RFP (5)* and *LSL-tdTomato* (3) mice were obtained from The Jackson Laboratory. All experimental mice were bred to a mixed CD1 albino background. Mice from experimental and control groups were randomly selected from either sex for live imaging experiments, and were within an adult age range of 2–4 months. The experiments were not randomized, and all experimentation involving animals were performed under the approval of the Yale School of Medicine Institutional Animal Care and Use Committee (IACUC).

In vivo imaging

Imaging procedures were adapted from those previously described (6). An isoflurane chamber was used to anesthetize mice, and then the mice were transferred to the imaging stage on a 37 °C heating pad. Mice were maintained on anesthesia throughout the course of recording with vaporized isoflurane delivered by a nose cone (1.25% in oxygen and air). The right paw was mounted on a custom-made stage and a glass coverslip was placed directly against the flat part of the paw. Image stacks were acquired with a LaVision TriM Scope II (LaVision Biotec) laser scanning microscope with a Chameleon Vision II (Coherent) two-photon laser (using 940 nm for live imaging) and a Chameleon Discovery (Coherent) two-photon laser (using 1120 nm for live imaging). A Nikon 25x/1 water immersion objective was used. Optical sections were scanned with a field of view of 0.3 x 0.3 mm^2. For all timelapse movies, the live mouse remained anesthetized for the length of the experiment and serial optical sections (3 µm steps for a total stack of 12 µm) were captured at intervals of 3.44 seconds for a total of 200 (11 minutes 28 seconds for topical pharmacological experiments) or 300 frames (17 minutes 12 seconds total recording time).

For revisits, the same region of live mouse skin was imaged across intervals of multiple days, and anatomical features of the paw, such as mouse digits, were used as landmarks for finding the same location.

Image analysis

Raw image stacks were imported into FIJI (ImageJ, National Institutes of Health) for analysis. Max or average (to select for persistently active behaviors specifically) Z stacks of sequential optical sections were generated. Translational motion correction of timelapse movies was performed in Imaris software.

Analysis of Ca^{2+} for individual ECs: Segmentation of the H2B-mCherry signal was performed via the threshold and masking functions on FIJI. Through a custom MATLAB pipeline, the GCaMP6s fluorescence intensity of each masked region of interest (ROI as a proxy for a cell) every 100 frames was normalized to the minimum fluorescence intensity over that period (Figure S2). Any change in fluorescence over a threshold (50% increase above the minimum fluorescence intensity) was treated as a Ca^{2+} event, and the duration of each event, as well as the number of events per ROI was calculated (Figure S2). To determine persistent signaling, a 50% change in minimum fluorescence intensity cannot be used because the mean MFI within the cell remains very

high for the duration of imaging. In this case, any EC with an MFI exceeding 170 (out of a maximum of 255), was treated as signaling for the duration it exceeded that threshold. Frequency is described as the number of events per minute, with the lowest possible frequency by cell of 0.0588 events/minute (1 event over the 17 minutes 12 seconds recording duration) or 0.001 Hz. Average duration of Ca^{2+} events was analyzed per EC by dividing total signaling duration by the number of Ca^{2+} events per cell.

Analysis of Ca^{2+} for EC clusters: Segmentation of the GCaMP6s signal with manual masking was used to segment the vessel surface. Using our custom MATLAB pipeline, we divided the masked vessel surface into individual grids, 0.0076 x 0.0076 mm^2 and the fluorescence intensity of each grid ROI was normalized every 100 frames by taking the minimum fluorescence value over each period (Figure S2). Any change in GCaMP6s fluorescence over 50% of the minimum fluorescence intensity was identified as a Ca^{2+} event, and the duration of each event, as well as the number of events per ROI was calculated. To analyze EC clusters, adjacent grid ROIs that displayed Ca^{2+} events either at the same frame or one frame apart were considered part of the cluster. The maximum number of grids with connected activity was represented, and the H2B-mCherry segmented ROIs were added to each set of connected grids to determine the number of ECs involved in each cluster (Figure S2).

Analysis and visualization of Ca^{2+} events by cell: A custom MATLAB pipeline was generated with assistance from ChatGPT-5 that takes an Excel spreadsheet of per-cell signaling event intervals, converts the event timing into seconds using the user-supplied frame time, and builds a binary cell × time activity raster showing where each cell is “on” during its event windows. The pipeline output was validated against cell signaling durations calculated manually to ensure the pipeline was functioning as anticipated. The pipeline then generates three visualizations labeled in seconds: (1) a raster heatmap of on/off signaling over time, (2) stacked per-cell “wave” traces, and (3) a bar plot of per-cell event frequency.

Analysis of vessel architecture: The capillary plexus in Cx43cKO mice was imaged with fine serial optical sections (0.5 µm steps for a total stack of 40 µm) on Day 0 and 14. The max projection of the optical sections was then used for subsequent analysis. A custom MATLAB script was generated with assistance from ChatGPT-5 to automatically identify fully enclosed vascular loops from a binary vessel mask. Each loop was labeled with a unique ID and its area, perimeter, centroid, and equivalent diameter were measured using MATLAB’s *regionprops* function. The pipeline was validated against regions where the parameters of loops were manually calculated.

Cell density: Endothelial cells were imaged with fine serial optical sections of 0.5 µm steps across 20-25 µm, on Day 0 and 14. Image stacks with uniform illumination across the scan area were chosen for analysis and were maximally projected for further segmentation. Nuclear masks were generated from the projected stacks using Cellpose-SAM (Stringer and Pachitaru, 2025) while the vascular skeleton was generated from a custom Python script. Each masked endothelial nuclear centroid was snapped onto the skeletonized vascular mask using a Euclidean distance transform and nuclei were retained only if the snapping distance was under 10 microns. Vessel segments were then detected as individual skeletonized paths between either branch points or endpoints using a connected neighborhood criterion and the length of each segment was calculated as a geodesic distance. For every segment, segment-wise linear endothelial cell density was then computed across the Day 0 and Day 14 revisits.

Line scan analysis: A custom MATLAB pipeline was generated with assistance from ChatGPT-5 that uses changes in MFI to understand cell flow (since cells are unlabeled in a TRITC dextran, black lines or MFI intensity valleys are cells moving through the dextran). The pipeline loads a RED 1-D intensity profile from a CSV (with user-selected columns if needed), prompts the user for peak/valley detection and block parameters, then identifies peaks and intensity-qualified valleys using prominence/distance/width rules. It converts the RED distance axis to a 0-based

coordinate system, partitions the profile into fixed-width blocks (e.g., 600 pixels), and for each block counts valleys and computes valley-to-valley spacings. Next, it loads a GREEN MFI CSV, averages GREEN MFI within the same RED-defined blocks, and automatically labels each block as no event/before/during/after based on whether block-mean MFI exceeds a threshold (1.5× the minimum block mean). The script then generates an overall figure with blocks shaded by event label and valleys overlaid, optionally exports per-block PNGs, and writes an Excel workbook containing summary, distances, and event legend outputs. For each vessel, five random 2-second time blocks (out of 150 total) were manually checked with the pipeline output to ensure the user selected parameters accurately capture intensity valleys (cells).

Average flux was calculated as the average flow rate during an individual signaling or non-signaling time period. Vessel flux represents a smoothed trend of vessel flow rate per time point, averaged over each time point and its neighboring 4 time points (2 prior and 2 after). Change in flow rate during a time period was calculated by looking at the difference in flow rates between the first and last time points during that period.

Quantitative PCR

RNA from isolated ECs was extracted using Qiagen RNeasy Plus Micro kit (74034). cDNA was made using SuperScript IV First-Strand Synthesis kit (Thermo Fisher 18091050). qPCR utilized FastStart Universal SYBR green Master (Sigma) on the CFX Connect Real-Time PCR Detection System (Bio-Rad). The set of primers used are as follows:

Connexin 37: forward, CCCACATCCGATACTGGGTG; reverse, CGAAGACGACCGTCCTCTG.

Connexin 40: forward, AGGGCTGAGCTTGCTTCTTA; reverse, TTAGTGCCAGTGTCGGGAAT.

Connexin 43: forward, GGTGATGAACAGTCTGCCTTTCG; reverse, GTGAGCCAAGTACAGGAGTGTG.

CACNA1C: forward, CGTTCTCATCCTGCTCAACACC; reverse, GAGCTTCAGGATCATCTCCACTG.

CACNA2D1: forward, GTGGAAGTGTGAGCGGATTGAC; reverse, TCGCTTGAACCAGGTGCTGGAA.

Tamoxifen induction

To induce the expression of GCaMP6s, H2B-mCherry, and/or loss of Cx43 expression, *VECadCreER; Rosa26-CAG-LSL-GCaMP6s; LSL-H2B-mCherry* or *VECadCreER; Cx43$^{fl/fl}$; Rosa26-CAG-LSL-GCaMP6s; LSL-H2B-mCherry* or *VECadCreER; Cx43$^{fl/fl}$; Rosa26-CAG-LSL-GCaMP6s* or *VECadCreER; Rosa26-CAG-LSL-GCaMP6s* or *VeCadCreER; Cx43$^{fl/fl}$* or *VeCadCreER; LSL-tdtomato* or *VeCadCreER* mice were given four doses of tamoxifen (2 mg in corn oil) 4, 5, 6, and 7 days before imaging or tissue collection by intraperitoneal injection (IP).

Topical drug treatment

For each drug tested, 4-6 capillary regions were imaged for 200 frames (11 minutes 28 seconds). To inhibit T-type VGCCs, TRPV4, or L-type VGCCs, mibefradil, GSK219, verapamil, and nifedipine respectively were delivered topically to the paw skin. Nifedipine, verapamil, and GSK219 were dissolved in a 100 mg ml^{-1} stock solution in dimethyl sulfoxide (DMSO) and 30 µl of the mixture was spread evenly on the paw for 15 minutes. Mibefradil was dissolved in a 50 mg ml^{-1} stock solution in dimethyl sulfoxide (DMSO) and 50 µl of the mixture was spread evenly on the

paw for 15 minutes. For lower doses of nifedipine treatment, the stock solution was diluted 10 or 100 times in DMSO before application. Regions in the paw were then revisited 30-60 minutes after application of the topical agents. A solution of 100% DMSO was used as vehicle control.

Capillary Visualization in aSMA-RFP mice

For visualization of capillaries in aSMA-RFP mice, 15 mg/kg of 150 kDa fluorescein isothiocyanate (FITC) dextran was injected retro-orbitally, and a 0.28 x 0.28 mm^2 region was imaged with a 40x water immersion objective, for a total depth of 50 microns with fine 0.5 µm steps. The max projection of 40 steps, or 20 µm of depth, was taken to capture the entire superficial capillary region.

Vascular permeability

A 0.3 x 0.3 mm^2 region was imaged for 100 frames (5 minutes 44 seconds) in *VECadCreER; Cx43$^{fl/fl}$; Rosa26-CAG-LSL-GCaMP6s;* or *VECadCreER; Rosa26-CAG-LSL-GCaMP6s* anesthetized mice prior to dextran injection. 15 mg/kg of tetramethylrhodamine (TRITC) dextran (70 kDa or 150 kDa molecular weight) was injected retro-orbitally into each mouse. 10-15 seconds after retroorbital injection, the dextran flow was recorded in the same 0.3 x 0.3 mm^2 region imaged before dextran injection, for a period of 100 frames using the Chameleon Discovery (Coherent) two-photon laser (1120 nm). For permeability rescue experiments, DMSO (vehicle) or nifedipine was spread evenly on the paw for 15 minutes, and then 15 mg/kg of TRITC-dextran (70 kDa molecular weight) was injected retro orbitally into each mouse.

For analysis of interstitial dextran leakage, segmentation of the TRITC-dextran with manual masking was used to segment the vascular structure. Pre-dextran, the GCaMP6S signal was used to segment the vessel surface. MFI of the regions outside the segmented vessel surface were calculated for each frame after dextran injection and normalized to the average extravascular MFI in the pre-dextran recording.

Vascular flow and line scanning

A 0.3 x 0.3 mm^2 region was imaged for 100 frames (5 minutes 44 seconds) in *VECadCreER; Cx43$^{fl/fl}$; Rosa26-CAG-LSL-GCaMP6s;* or *VECadCreER; Rosa26-CAG-LSL-GCaMP6s* anesthetized mice prior to dextran injection. 15 mg/kg of TRITC-dextran (150 kDa molecular weight) was injected retro-orbitally into each mouse. Line scanning was conducted in the middle of individual vessels, at a frequency of 600 line scans over one optical section across each 2 second period. Each vessel was scanned for 150 time steps, or 300 seconds total. Vessels with large changes in baseline MFI during tracking were discarded for analysis, as this was an indication that the FOV shifted such that the vessel was not being captured throughout the imaging timeframe. Capillary vessels were chosen across the superficial plexus and were not classified based on architectural features and relation to deeper plexi. We imaged and assessed flow in the center of 20 vessels per mouse across multiple regions of the superficial capillary plexus (10-15% of capillary segments per region).

For flow rate rescue experiments, DMSO (vehicle) or nifedipine was spread evenly on the paw for 15 minutes, and then 15 mg/kg of TRITC-dextran (70 kDa molecular weight) was injected retro orbitally into each mouse prior to line scanning for 30-60 minutes after exposure.

Endothelial cell isolation and sorting

VECadCreER, VECadCreER; LSL-tdtomato, and *VECadCreER; Cx43$^{fl/fl}$* mice were induced with tamoxifen 1 week prior to harvesting tissue. Adult paws were collected and placed dermis side down in a 5 mg/ml dispase II solution (MilliporeSigma; 494207800) for 45 minutes at 37 °C. The epidermis was removed, and the dermis was minced finely and transferred to 0.25% collagenase IV (Sigma; C5138) in HBSS (GIBCO;14170-112) solution for at least 90 minutes at 37 °C. The tissue was then passed through an 18-gauge needle (BD; 305195) and washed in FACS Buffer (3% FBS, 2mM EDTA). The suspensions were then centrifuged at 350G for 10 minutes and filtered through a 40 µm filter (Falcon; 352340) before staining with anti-mouse CD31 APC

(Biolegend;160210; 1/200) for sorting on a BD FACS AriaII. ECs were sorted based on expression of CD31 (and for *tdtomato* in *VECadCreER; LSL-tdtomato mice*). *aSMA-RFP* mice were used for harvesting tissue without any tamoxifen exposure, and the RFP was used to isolate cells.

Statistics and reproducibility

GraphPad Prism software (GraphPad, Inc.) was used to perform statistical analyses (version 9.2). Parameters are reported in the figure legends, and comparisons between groups were made using paired or unpaired two-tailed Student's *t* test (in short: t-test), or Chi-squared analysis for observed versus expected outcomes (df=1) in revisits. Normality was assumed in data distribution, but not formally tested. Differences between groups were considered significant at $P < 0.05$ and the data are presented as means ± SD.

High frequency or high average duration EC populations were designated as displaying activity 1 standard deviation (SD) greater than the mean of all active ECs. For control revisits, the value threshold for >1SD dynamics cells was calculated based on frequency and average duration distributions on Day 0. For Cx43cKO mice, the value threshold for >1SD dynamics cells was based on the frequency and average duration distributions of the control mice.

For Chi-squared analysis, expected values of ECs active on Day 0 and then also when revisited ('random activity' as the null hypothesis) were assigned based on the percentage of ECs displaying activity on Day 0 (58% for 24-hour revisits, 54% for 14-day revisits, and 74% for Cx43cKO 14-day revisits). These proportions were then compared to the percentage of cells that were observed to maintain their activity status upon revisiting.

To assess changes in frequency and average duration dynamics in revisited regions after pharmacological application of DMSO or nifedipine, the mean values of frequency and average duration were calculated for each mouse before and after treatment, and the percentage change between the pre-treatment and post-treatment values was compared between the two conditions.

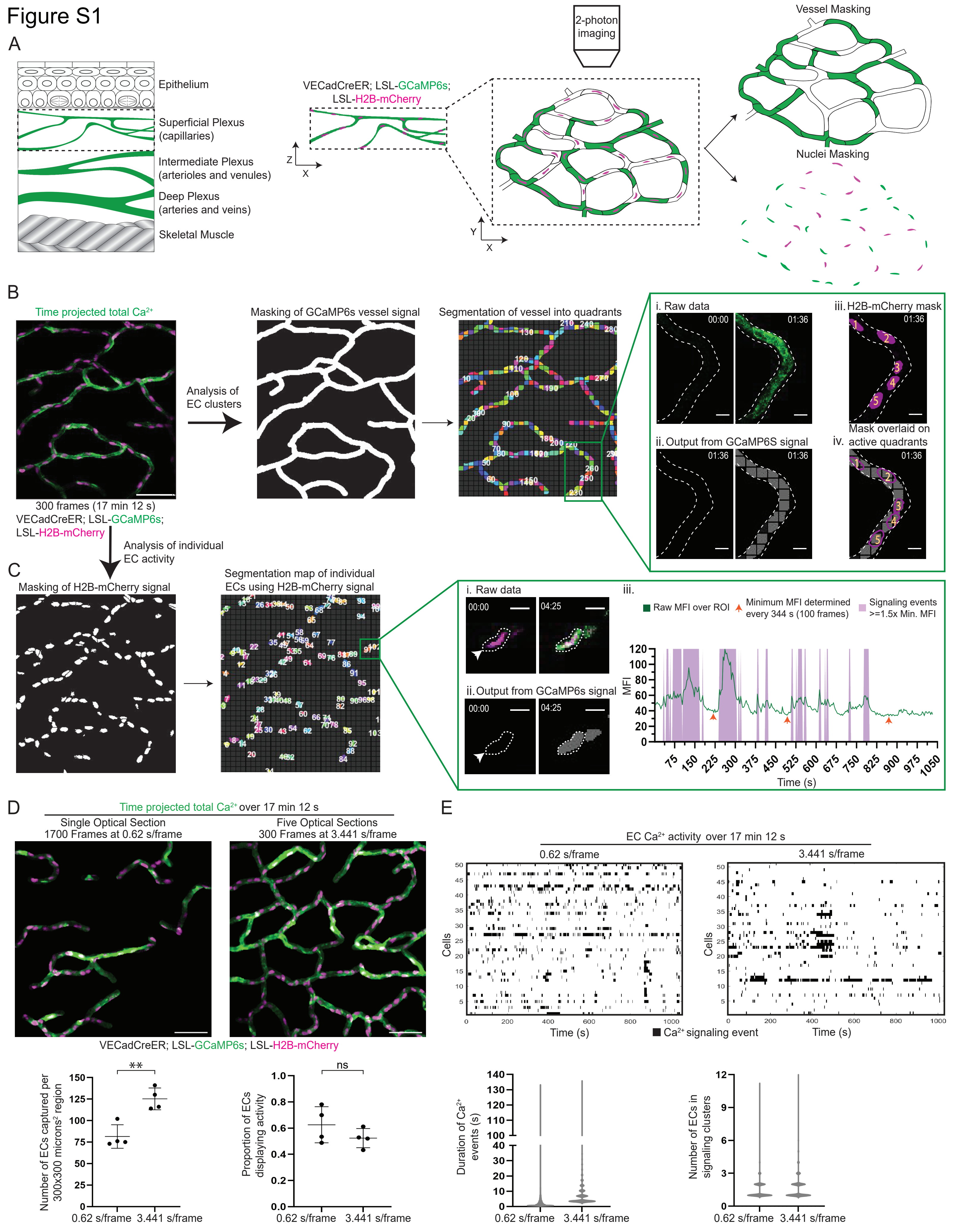

Figure S1
A
Epithelium
Superficial Plexus (capillaries)
Intermediate Plexus (arterioles and venules)
Deep Plexus (arteries and veins)
Skeletal Muscle
VECadCreER; LSL-GCaMP6s; LSL-H2B-mCherry
2-photon imaging
Vessel Masking
Nuclei Masking
B
Time projected total Ca2+
Analysis of EC clusters
Masking of GCaMP6s vessel signal
Segmentation of vessel into quadrants
i. Raw data
iii. H2B-mCherry mask
ii. Output from GCaMP6S signal
iv. Mask overlaid on active quadrants
300 frames (17 min 12 s)
VECadCreER; LSL-GCaMP6s; LSL-H2B-mCherry
Analysis of individual EC activity
C
Masking of H2B-mCherry signal
Segmentation map of individual ECs using H2B-mCherry signal
i. Raw data
ii. Output from GCaMP6s signal
iii.
Raw MFI over ROI
Minimum MFI determined every 344 s (100 frames)
Signaling events >=1.5x Min. MFI
MFI
Time (s)
D
Time projected total Ca2+ over 17 min 12 s
Single Optical Section 1700 Frames at 0.62 s/frame
Five Optical Sections 300 Frames at 3.441 s/frame
VECadCreER; LSL-GCaMP6s; LSL-H2B-mCherry
Number of ECs captured per 300x300 microns2 region
Proportion of ECs displaying activity
0.62 s/frame
3.441 s/frame
**
ns
E
EC Ca2+ activity over 17 min 12 s
0.62 s/frame
3.441 s/frame
Cells
Time (s)
Ca2+ signaling event
Duration of Ca2+ events (s)
Number of ECs in signaling clusters

Figures

Fig. S1. Schematic of intravital imaging and analysis platforms in Ca^{2+} sensor mice and analysis across kinetic scales (A) *Left*: Cartoon representing skin in XZ plane, outlining the superficial capillary plexus. *Middle*: Cartoon of 2-photon imaging over the XY plane of the capillary region, with EC-specific GCaMP6s signal (green) and H2B-mCherry signal (magenta). *Right*: Representation of vessel and nuclei masking from imaged regions. **(B)** *Left:* Max intensity projection of GCaMP6s signal (green) with H2B-mCherry signal (magenta) from 300 frame (17 minutes 12 seconds) recording of skin capillary ECs (scale bar: 50 μm). *Middle:* Masking of vessels based on GCaMP6s signal, and segmentation of vessel mask into 0.0076 x 0.0076 mm^2 quadrants. *Right:* **(i)** Representative images of a Ca^{2+} event, with the **(ii)** pipeline output displaying the number of quadrants involved (scale bar: 10 μm). An event is defined as at least a 50% increase above mean fluorescence intensity (MFI) over the minimum fluorescence for each quadrant. **(iii)** and **(iv)** Adding the H2B-mCherry signal over the pipeline output allows determining the number of ECs involved. **(C)** *Left:* Masking of nuclei based on H2B-mCherry signal as a proxy for individual ECs *Middle:* Segmentation of nuclei mask into individual regions of interest (ROIs) *Right:* **(i)** Representative images of a Ca^{2+} event (scale bar: 10 μm), with the **(ii)** pipeline output displaying an event. An event is defined as at least a 50% increase above MFI over the minimum fluorescence for each ROI. **(iii)** MFI (green) over the ROI over 300 frames of recording (17 minutes 12 seconds), with minimum MFI calculated every 100 frames (orange arrowheads), and overlaid output Ca^{2+} events (purple). **(D)** *Top:* Max intensity projection over 17 minutes 12 seconds recording at two frame rates: 0.62 s/frame over a single optical section or 3.44 s/frame over 5 optical sections (scale bar: 50 μm). *Bottom Left:* Number of ECs captured per 0.3 x 0.3 mm^2 region when imaged at 0.62 s/frame and 3.44 s/frame. P=0.003, unpaired t-test; n=11 regions from 4 mice imaged at 0.62 s/frame and 14 regions from 4 mice imaged at 3.44 s/frame. *Bottom Right:* Proportion of active ECs in mice imaged at 0.62 s/frame and 3.44 s/frame. P=0.239, unpaired t-test; n=11 regions from 4 mice imaged at 0.62 s/frame and 14 regions from 4 mice imaged at 3.44 s/frame. **(E)** *Top:* Representative plots of Ca^{2+} signaling events (black) for 50 active ECs imaged at 0.62 s/frame and 50 active ECs imaged at 3.44 s/frame. *Bottom:* Duration of Ca^{2+} events and number of ECs in signaling clusters in mice imaged at 0.62 s/frame and 3.44 s/frame; n=8,733 events from 4 mice imaged at 0.62 s/frame and 6,936 events from 4 mice imaged at 3.44 s/frame.

Figure S2

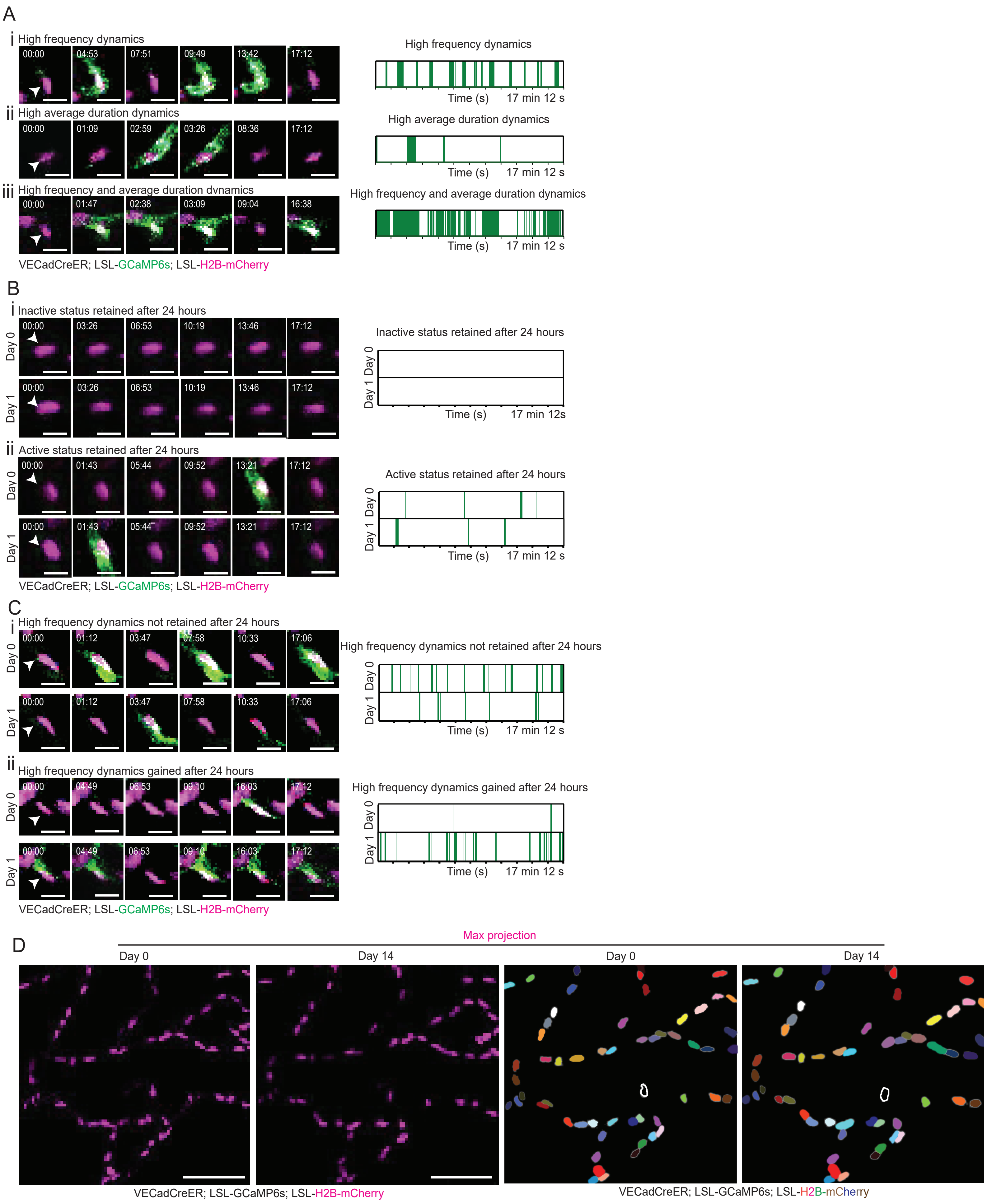
A
i High frequency dynamics
00:00
04:53
07:51
09:49
13:42
17:12
High frequency dynamics
Time (s)
17 min 12 s
ii High average duration dynamics
00:00
01:09
02:59
03:26
08:36
17:12
High average duration dynamics
Time (s)
17 min 12 s
iii High frequency and average duration dvnamics
00:00
01:47
02:38
03:09
09:04
16:38
High frequency and average duration dynamics
Time (s)
17 min 12 s
VECadCreER; LSL-GCaMP6s; LSL-H2B-mCherry
B
i Inactive status retained after 24 hours
Day 0
Day 1
00:00
03:26
06:53
10:19
13:46
17:12
Inactive status retained after 24 hours
Time (s)
17 min 12s
ii Active status retained after 24 hours
00:00
01:43
05:44
09:52
13:21
17:12
Active status retained after 24 hours
Time (s)
17 min 12 s
VECadCreER; LSL-GCaMP6s; LSL-H2B-mCherry
C
i High frequency dynamics not retained after 24 hours
00:00
01:12
03:47
07:58
10:33
17:06
High frequency dynamics not retained after 24 hours
Time (s)
17 min 12 s
ii High frequency dynamics gained after 24 hours
00:00
04:49
06:53
09:10
16:03
17:12
High frequency dynamics gained after 24 hours
Time (s)
17 min 12 s
VECadCreER; LSL-GCaMP6s; LSL-H2B-mCherry
D
Max projection
Day 0
Day 14
Day 0
Day 14
VECadCreER; LSL-GCaMP6s; LSL-H2B-mCherry
VECadCreER; LSL-GCaMP6s; LSL-H2B-mCherry

Fig. S2. Ca^{2+} signaling dynamics and EC positional stability over time (A) (i) Representative image of EC (nuclei in magenta) displaying high frequency dynamics with plot showcasing Ca^{2+} events (green) and their durations over 300 frames (17 minutes 12 seconds) of recording (scale bar: 10 μm). **(ii)** EC displaying high average duration dynamics. **(iii)** EC displaying high frequency and high average duration dynamics. **(B) (i)** Representative image of EC displaying inactive status on Day 0 and again when revisited 24 hours later, (scale bar: 10 μm). **(ii)** EC displaying activity on Day 0 and again when revisited 24 hours later. **(C) (i)** Representative image of EC losing high frequency dynamics when revisited 24 hours later. (scale bar: 10 μm). **(ii)** EC gaining high frequency dynamics when revisited 24 hours later. (**D**) *Left:* Representative image of region revisited on Day 0 and Day 14 (scale bar: 50 μm). *Right:* ECs multi-color coded, with the same colors on Day 0 and Day 14 corresponding to the same EC.

Figure S3

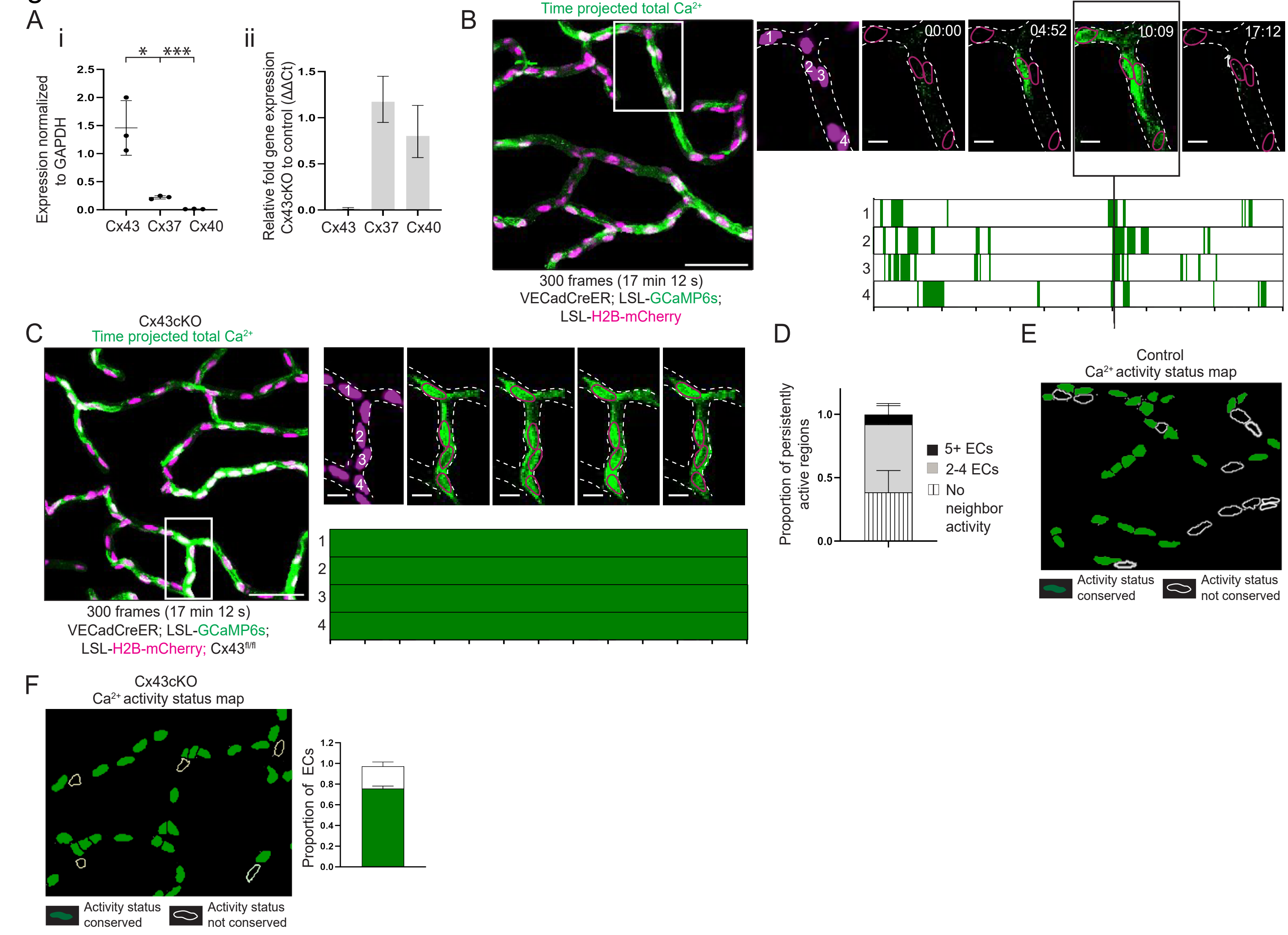

A
i
ii
Expression normalized to GAPDH
Cx43 Cx37 Cx40
Relative fold gene expression Cx43cKO to control (ΔΔCt)
B
Control
Time projected total Ca2+
00:00
04:52
10:09
17:12
300 frames (17 min 12 s)
VECadCreER; LSL-GCaMP6s;
LSL-H2B-mCherry
C
Cx43cKO
Time projected total Ca2+
300 frames (17 min 12 s)
VECadCreER; LSL-GCaMP6s;
LSL-H2B-mCherry; Cx43fl/fl
D
Proportion of persistently active regions
5+ ECs
2-4 ECs
No neighbor activity
E
Control
Ca2+ activity status map
Activity status conserved
Activity status not conserved
F
Cx43cKO
Ca2+ activity status map
Proportion of ECs
Activity status conserved
Activity status not conserved

Fig. S3. Spatial and molecular analyses of Ca^{2+} dynamics across control and Cx43cKO mice **(A) (i)** Expression of vascular connexins (Cx43, Cx37, and Cx40) in sorted skin ECs, normalized to GAPDH expression. *n*= 3 mice. **(ii)** Relative expression ($\Delta\Delta C_t$ method) of vascular connexins after Cx43cKO, compared to control mice. Connexin expression in Cx43cKO mice is first normalized to GAPDH, and then to expression in control mice. *n*= 3 Cx43cKO 3 control mice. (**B**) *Left*: Max intensity projection of GCaMP6s signal (green) with H2B-mCherry signal (magenta) from 300 frame (17 minutes 12 seconds) recording of skin capillary ECs in control mice (scale bar: 50 µm). *Right*: Inset of a region with Ca^{2+} activity occurring simultaneously across 4 ECs (numbered and drawn in magenta outline matching H2B-mCherry) (scale bar: 10 µm). *Bottom*: Ca^{2+} events and their durations over recording time for each EC. Black line across plots indicates the timepoint when all 4 ECs simultaneously display Ca^{2+} activity. **(C)** *Left*: GCaMP6s and H2B-mCherry signal from skin capillary ECs in Cx43cKO mice (scale bar: 50 µm). *Right*: Inset of a region with Ca^{2+} activity occurring simultaneously across 4 persistently active ECs (scale bar: 10 µm). *Bottom*: Ca^{2+} events for each persistently active EC. **(D)** Proportion of persistently active regions involving different EC cluster sizes. *n* = 10 regions from 3 mice. **(E)** Ca^{2+} activity status map for control ECs revisited after 14 days. Non-conserved activity status (white outline) and conserved activity status (green). *n*= 4 regions from 3 mice. **(F)** Ca^{2+} activity status map for Cx43cKO ECs revisited after 14 days. Proportion of ECs by their conservation of activity status. Chi-square analysis P<0.0001. *n*= 7 regions from 4 mice.

Figure S4

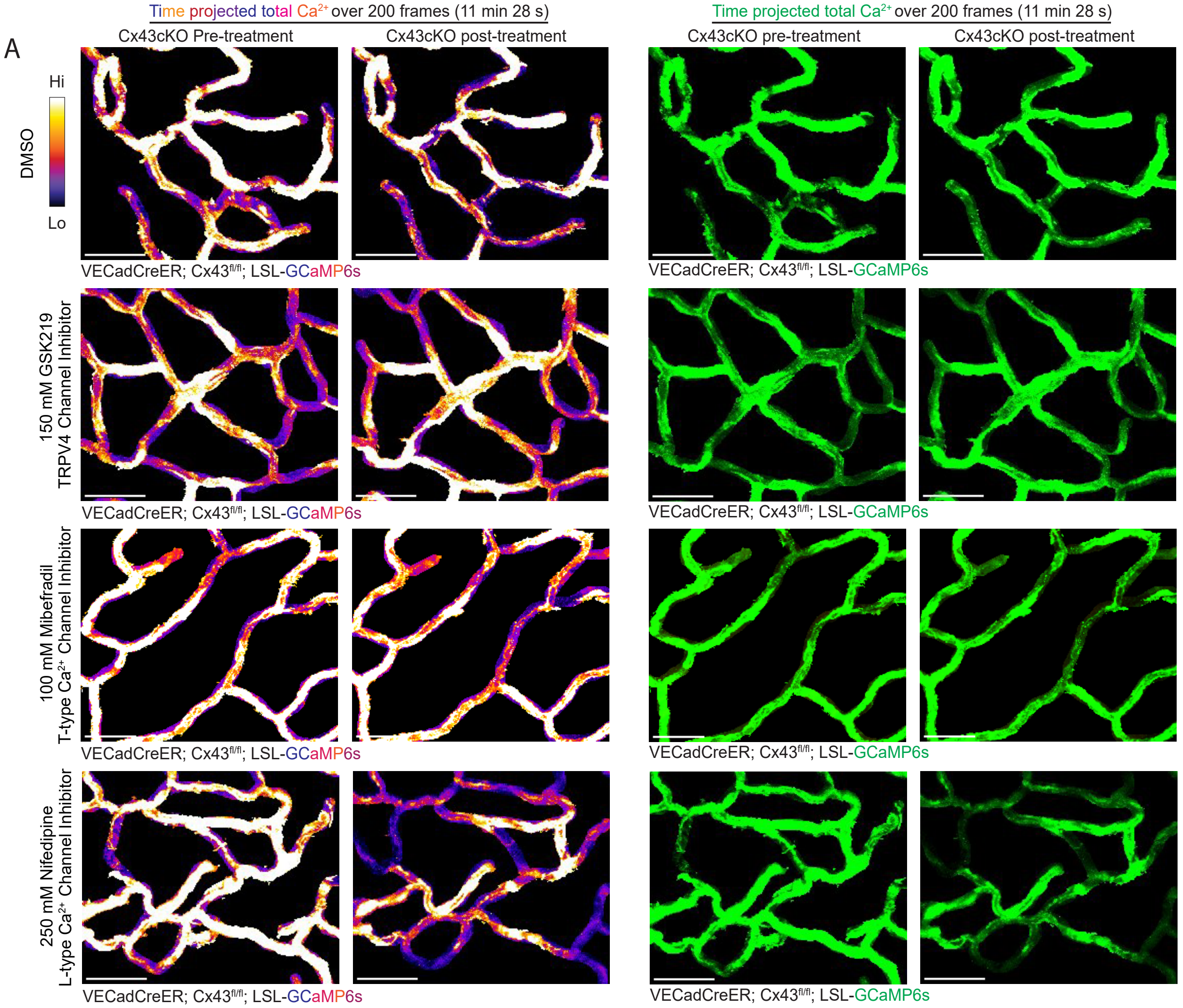
Time projected total Ca2+ over 200 frames (11 min 28 s)
Cx43cKO Pre-treatment
Cx43cKO post-treatment
Time projected total Ca2+ over 200 frames (11 min 28 s)
Cx43cKO pre-treatment
Cx43cKO post-treatment
A
Hi
DMSO
Lo
VECadCreER; Cx43fl/fl; LSL-GCaMP6s
VECadCreER; Cx43fl/fl; LSL-GCaMP6s
150 mM GSK219
TRPV4 Channel Inhibitor
VECadCreER; Cx43fl/fl; LSL-GCaMP6s
VECadCreER; Cx43fl/fl; LSL-GCaMP6s
100 mM Mibefradil
T-type Ca2+ Channel Inhibitor
VECadCreER; Cx43fl/fl; LSL-GCaMP6s
VECadCreER; Cx43fl/fl; LSL-GCaMP6s
250 mM Nifedipine
L-type Ca2+ Channel Inhibitor
VECadCreER; Cx43fl/fl; LSL-GCaMP6s
VECadCreER; Cx43fl/fl; LSL-GCaMP6s

Fig. S4. L-type VGCC inhibition, relative to inhibiting T-type VGCCs, and TRPV4, decreases Ca^{2+} activity after loss of Cx43 (A) Max intensity projection of GCaMP6s signal in green and fire lookup table from 200 frame (11 minutes 28 seconds) recording of skin capillary ECs in Cx43cKO mice before and after treatment with DMSO, GSK219, Mibefradil, and Nifedipine. Fire lookup table allows for easier visualization of changes in Ca^{2+} signaling intensity. *n*= 3 mice for each condition (scale bar: 50 μm).

Figure S5

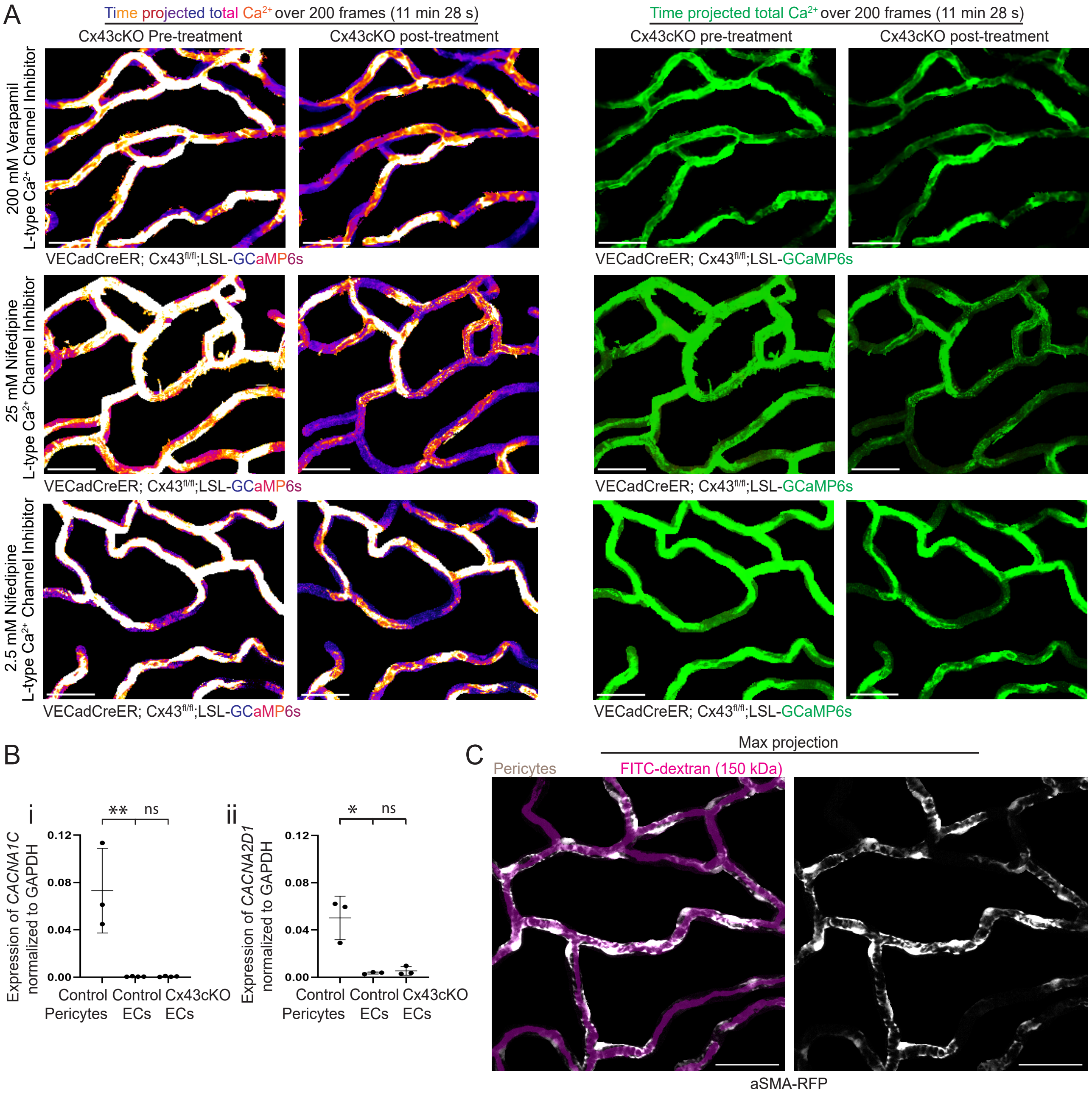
A
Time projected total Ca2+ over 200 frames (11 min 28 s)
Cx43cKO Pre-treatment
Cx43cKO post-treatment
Time projected total Ca2+ over 200 frames (11 min 28 s)
Cx43cKO pre-treatment
Cx43cKO post-treatment
200 mM Verapamil L-type Ca2+ Channel Inhibitor
25 mM Nifedipine L-type Ca2+ Channel Inhibitor
2.5 mM Nifedipine L-type Ca2+ Channel Inhibitor
VECadCreER; Cx43fl/fl;LSL-GCaMP6s
B
i
ii
Expression of CACNA1C normalized to GAPDH
Expression of CACNA2D1 normalized to GAPDH
0.12
0.08
0.04
0.00
**
*
ns
Control Pericytes
Control ECs
Cx43cKO ECs
C
Max projection
Pericytes
FITC-dextran (150 kDa)
aSMA-RFP

Fig. S5. Inhibition of L-type VGCCs specifically decreases Ca^{2+} activity after loss of Cx43 through non-cell autonomous regulation (A) Max intensity projection of recording before and after treatment with verapamil, and nifedipine (10 times and 100 times lower dose). Fire lookup table allows for easier visualization of changes in Ca^{2+} signaling intensity. *n*= 3 mice for each condition (scale bar: 50 μm). **(B) (i)** Expression of *CACNA1C* in sorted skin ECs and smooth muscle actin (SMA+) pericytes during homeostasis, and skin ECs after loss of Cx43, normalized to GAPDH expression. P=0.009 and ns: P>0.05 unpaired t-tests respectively; *n*= 3 aSMA-RFP mice to isolate SMA+ pericytes, n=4 control and Cx43cKO mice. **(ii)** Expression of *CACNA2D1* normalized to GAPDH expression. P=0.0479 and ns: P>0.05 unpaired t-tests respectively; *n*= 3 aSMA-RFP mice, n= 4 control and Cx43cKO mice. **(C)** Representative images of capillary region (grey) in aSMA-RFP mice with 150 kDa FITC dextran (magenta) (scale bar: 50 μm).

Figure S6

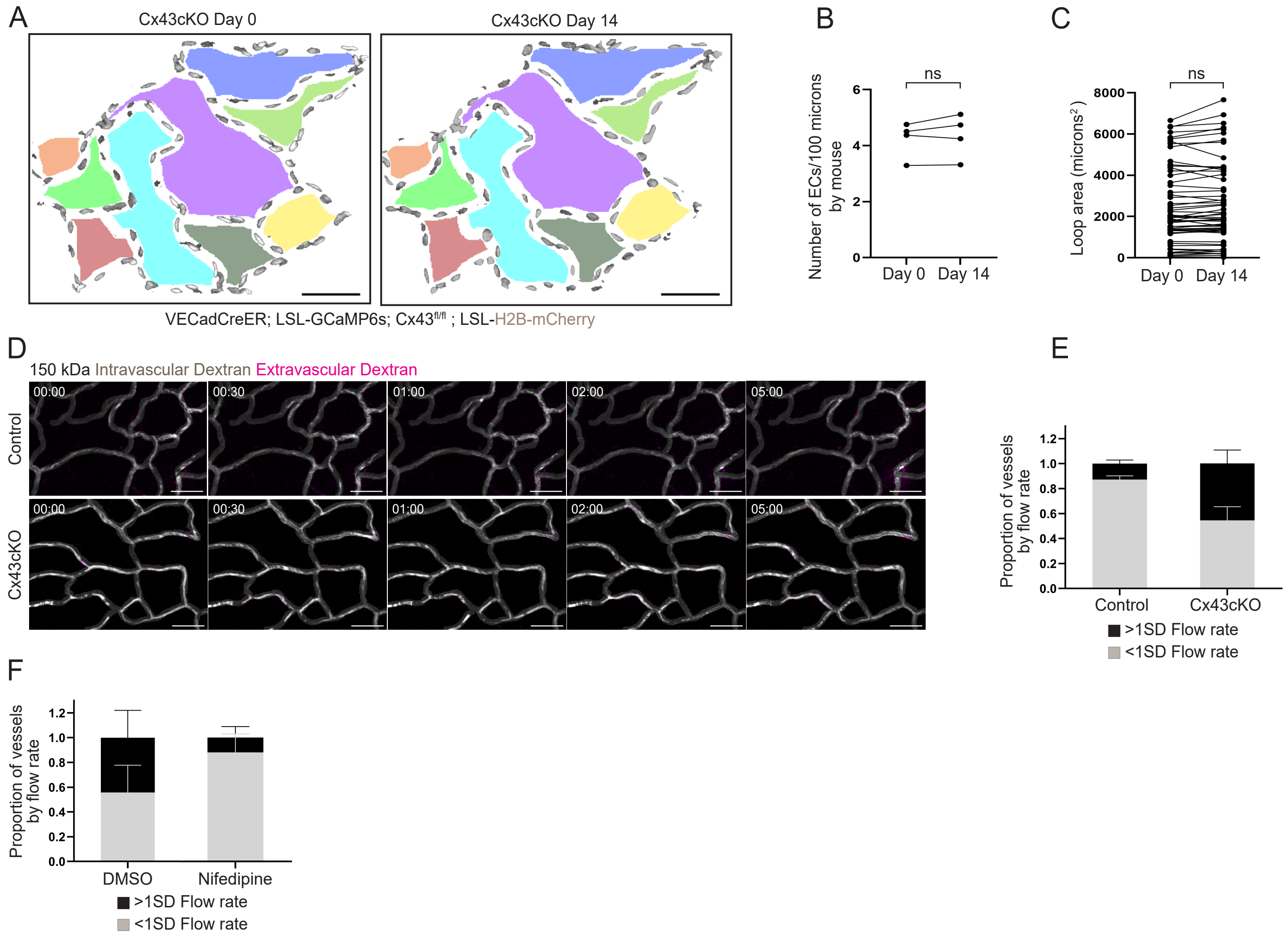

A
Cx43cKO Day 0
Cx43cKO Day 14
VECadCreER; LSL-GCaMP6s; Cx43fl/fl ; LSL-H2B-mCherry
B
ns
Number of ECs/100 microns by mouse
6
4
2
0
Day 0
Day 14
C
ns
Loop area (microns2)
8000
6000
4000
2000
0
Day 0
Day 14
D
150 kDa Intravascular Dextran Extravascular Dextran
Control
Cx43cKO
00:00
00:30
01:00
02:00
05:00
E
Proportion of vessels by flow rate
1.2
1.0
0.8
0.6
0.4
0.2
0.0
Control
Cx43cKO
>1SD Flow rate
<1SD Flow rate
F
Proportion of vessels by flow rate
1.2
1.0
0.8
0.6
0.4
0.2
0.0
DMSO
Nifedipine
>1SD Flow rate
<1SD Flow rate

Fig. S6. Architectural, barrier, and flow functional analyses downstream of Ca^{2+} elevation after loss of Cx43 (A) *Left:* Max intensity projection of capillary ECs in a Cx43cKO mouse with H2B-mCherry signal (gray) and color-coded architectural loops (scale bar: 50 µm). *Right:* Max intensity projection of the same cells revisited after 14 days, with the same colors corresponding to the same vessel loops (scale bar: 50 µm). **(B)** Number of ECs per 100 µm of the same regions revisited on Day 0 and Day 14 in Cx43cKO mice; ns: P>0.05, paired t-test, *n*=6 regions total from 4 mice. **(C)** Area of the same architectural loops revisited on Day 0 and Day 14 in Cx43cKO mice; ns: P>0.05, paired t-test, *n*=59 total vessel loops from 4 mice. **(D)** Representative single time point images of 150 kDa intravascular dextran (grey) with no extravascular dextran in magenta (scale bar: 50 µm). *n*= 3 mice each for control and Cx43cKO. **(E)** Proportion of vessels by their flow rate in control and Cx43ckO mice, separated by >1SD fast flow vessels (black) and <1SD flow vessels (grey). *n*=45 vessels from 3 mice for each group. **(F)** Proportion of vessels by their flow rate in Cx43cKO mice treated with DMSO or nifedipine. *n*=45 vessels from 3 mice for each group.

Figure S7

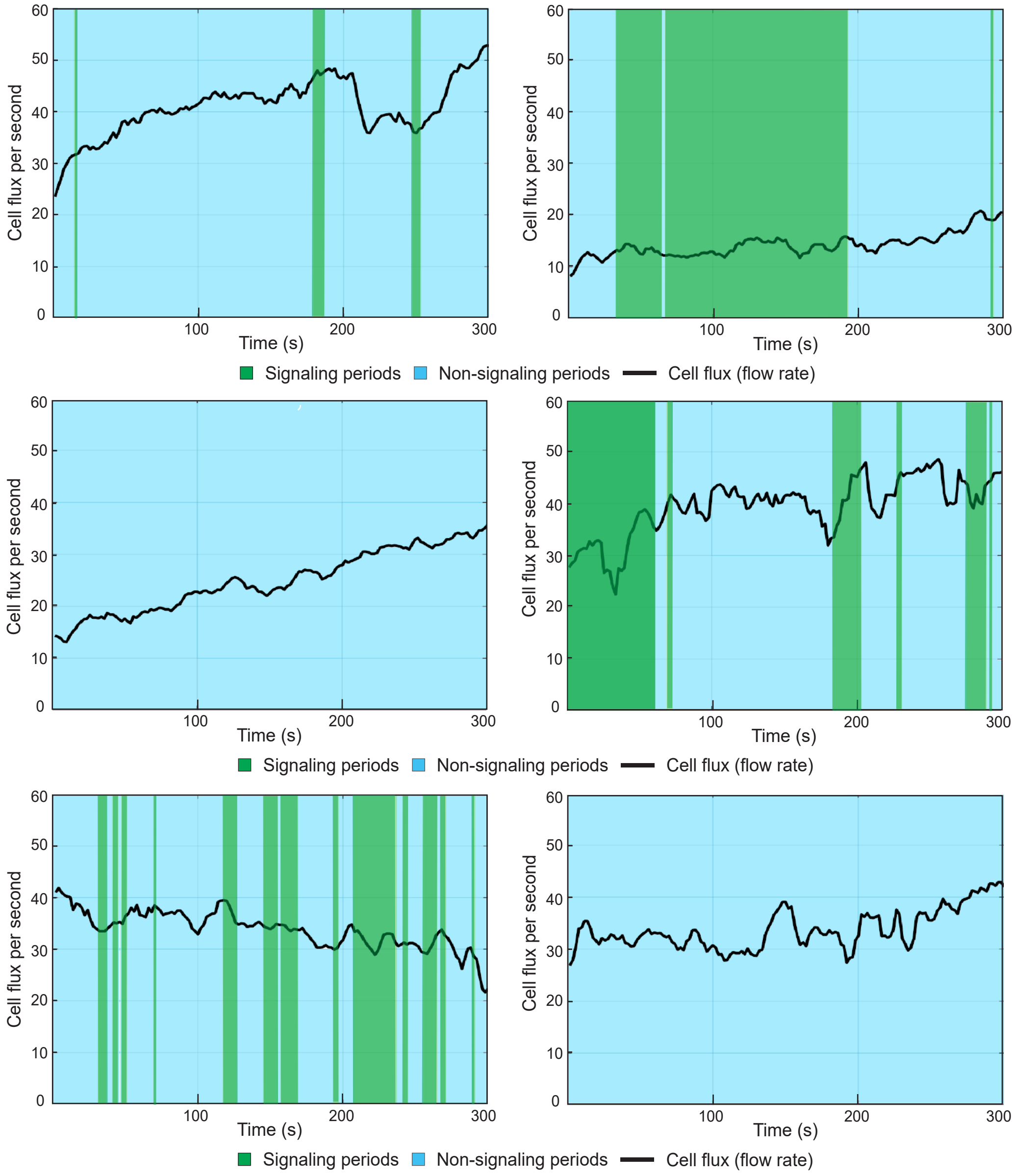

Cell flux per second
Time (s)
0
10
20
30
40
50
60
100
200
300
Signaling periods
Non-signaling periods
Cell flux (flow rate)

Fig. S7. Vessel flow and Ca^{2+} signaling during homeostasis (A) Example graphs across multiple vessels representing cell flux per second or flow rate during line scanning over a 5 min period. Flow rate (black line) is represented over signaling (green) and non-signaling (light blue) time periods.

Figure S8

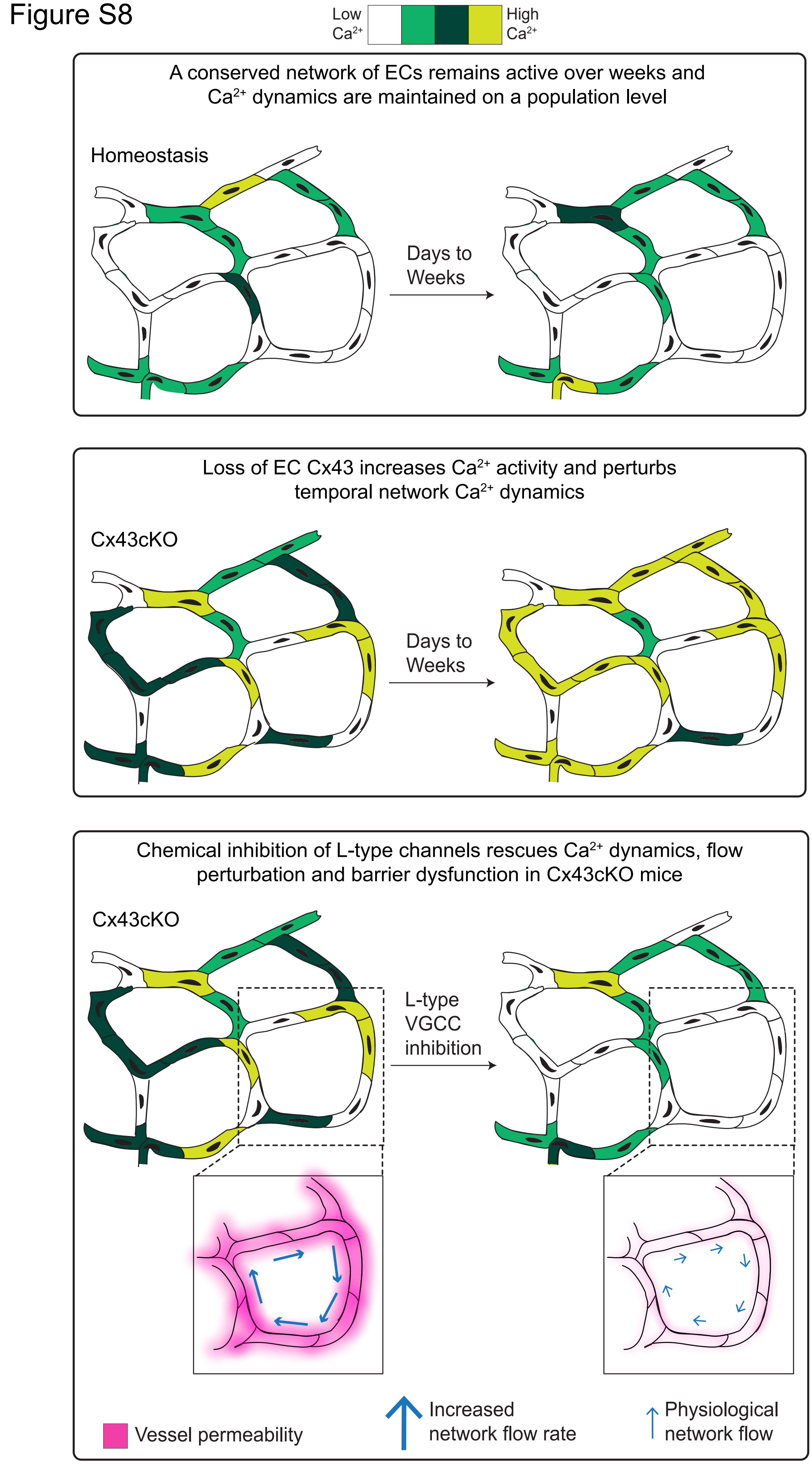

Low Ca2+
High Ca2+
A conserved network of ECs remains active over weeks and Ca2+ dynamics are maintained on a population level
Homeostasis
Days to Weeks
Loss of EC Cx43 increases Ca2+ activity and perturbs temporal network Ca2+ dynamics
Cx43cKO
Days to Weeks
Chemical inhibition of L-type channels rescues Ca2+ dynamics, flow perturbation and barrier dysfunction in Cx43cKO mice
Cx43cKO
L-type VGCC inhibition
Vessel permeability
Increased network flow rate
Physiological network flow

Fig. S8. A conserved network orchestrates Ca^{2+} patterning in capillary ECs over days to weeks and is spatiotemporally and functionally regulated by Cx43. Model depicting the spatiotemporal characteristics of Ca^{2+} signaling organization in capillary ECs. A spatiotemporally conserved network of ECs orchestrates tissue-wide Ca^{2+} activity over days to weeks and maintains Ca^{2+} dynamics at the population level. Loss of Cx43 increases Ca^{2+} activity and impairs long-term temporal regulation of the EC network, converging on a persistently active phenotype. Sustained Ca^{2+} activity leads to flow and barrier dysfunction. L-type VGCC inhibition non cell-autonomously restores Ca^{2+} activity, flow, and barrier function in Cx43cKO mice to physiological patterns.

Movie S1. EC Ca^{2+} activity in skin capillaries is widespread and heterogenous during homeostasis Timelapse of GCaMP6s signal (green) and H2B-mCherry (magenta) from 300 frame (17 minutes 12 seconds) recording of skin capillary ECs, imaged at a frame rate of 3.44 s/frame (scale bar: 20 µm). Timelapse is followed by a max intensity projection of GCaMP6s signal represented with fire lookup table. Color scale indicates GCaMP6s signal over recording time.

Movie S2. EC Ca^{2+} activity status is conserved on a single-cell level after 24 hours *Left*: Timelapse of GCaMP6s signal (green) and H2B-mCherry (magenta) from 300 frame (17 minutes 12 seconds) recording of skin capillary ECs on baseline Day 0, imaged at a frame rate of 3.44 s/frame (scale bar: 20 µm). *Right:* Timelapse of region from Day 0 revisited after 24 hours. Timelapse is followed by a max intensity projection of GCaMP6s signal for Day 0 and Day 1 represented with fire lookup table. Color scale indicates GCaMP6s signal over recording time.

Movie S3. EC Ca^{2+} activity status is conserved on a single-cell level after 14 days *Left*: Timelapse of GCaMP6s signal (green) and H2B-mCherry (magenta) from 300 frame (17 minutes 12 seconds) recording of skin capillary ECs on baseline Day 0, imaged at a frame rate of 3.44 s/frame (scale bar: 20 µm). *Right:* Timelapse of region from Day 0 revisited after 2 weeks. Timelapse is followed by a max intensity projection of GCaMP6s signal for Day 0 and Day 14 represented with fire lookup table. Color scale indicates GCaMP6s signal over recording time.

Movie S4. Cx43cKO leads to sustained EC Ca^{2+} activity *Left*: Timelapse of GCaMP6s signal (green) and H2B-mCherry (magenta) from 300 frame (17 minutes 12 seconds) recording of skin capillary ECs from control mice, imaged at a frame rate of 3.44 s/frame (scale bar: 20 µm). *Right:* Timelapse of capillary ECs from Cx43cKO mice. Timelapse is followed by a max intensity projection of GCaMP6s signal for control and Cx43cKO mice, represented with fire lookup table. Color scale indicates GCaMP6s signal over recording time.

Movie S5. Cx43cKO leads to increase in persistently active ECs after 2 weeks *Left*: Timelapse of GCaMP6s signal (green) and H2B-mCherry (magenta) from 300 frame (17 minutes 12 seconds) recording of skin capillary ECs from Cx43cKO mice on baseline Day 0, imaged at a frame rate of 3.44 s/frame (scale bar: 20 µm). *Right:* Timelapse of region from Day 0 revisited after 2 weeks, on Day 14. Timelapse is followed by an average intensity projection of GCaMP6s signal for Cx43cKO mice on Day 0 and Day 14, represented with fire lookup table. Average intensity projections allow better visualization of persistently active regions in white. Color scale indicates GCaMP6s signal over recording time.

Movie S6. L-type VGCC inhibition does not affect EC Ca^{2+} activity in control mice *Top Left*: Timelapse of GCaMP6s signal (green) and H2B-mCherry (magenta) from 200 frame (11 minutes 28 seconds) recording of skin capillary ECs from control mice prior to DMSO treatment, imaged at a frame rate of 3.44 s/frame (scale bar: 20 µm). *Top Right*: Timelapse of revisited region after DMSO treatment. *Bottom Left*: Timelapse of ECs prior to nifedipine treatment. *Bottom Right*: Timelapse of revisited region after nifedipine treatment. All timelapses are followed by max intensity projection of GCaMP6s signal represented with fire lookup table. Color scale indicates GCaMP6s signal over recording time.

Movie S7. L-type VGCC inhibition decreases EC Ca^{2+} activity after Cx43cKO *Top Left*: Timelapse of GCaMP6s signal (green) and H2B-mCherry (magenta) from 200 frame (11 minutes 28 seconds) recording of skin capillary ECs from Cx43cKO mice prior to DMSO treatment, imaged at a frame rate of 3.44 s/frame (scale bar: 20 µm). *Top Right*: Timelapse of revisited region after DMSO treatment. *Bottom Left*: Timelapse of ECs prior to nifedipine treatment. *Bottom Right*: Timelapse of revisited region after nifedipine treatment. All timelapses are followed by max intensity projection of GCaMP6s signal represented with fire lookup table. Color scale indicates GCaMP6s signal over recording time.

SI References

1. I. Sorensen, R. H. Adams, A. Gossler, DLL1-mediated Notch activation regulates endothelial identity in mouse fetal arteries. *Blood* **113**, 5680-5688 (2009).
2. Y. Liao, K. H. Day, D. N. Damon, B. R. Duling, Endothelial cell-specific knockout of connexin 43 causes hypotension and bradycardia in mice. *Proc Natl Acad Sci U S A* **98**, 9989-9994 (2001).
3. L. Madisen *et al.*, Transgenic mice for intersectional targeting of neural sensors and effectors with high specificity and performance. *Neuron* **85**, 942-958 (2015).
4. S. P. Peron, J. Freeman, V. Iyer, C. Guo, K. Svoboda, A Cellular Resolution Map of Barrel Cortex Activity during Tactile Behavior. *Neuron* **86**, 783-799 (2015).
5. V. S. LeBleu *et al.*, Identification of human epididymis protein-4 as a fibroblast-derived mediator of fibrosis. *Nat Med* **19**, 227-231 (2013).
6. C. M. Pineda *et al.*, Intravital imaging of hair follicle regeneration in the mouse. *Nat Protoc* **10**, 1116-1130 (2015).
